# Electrocatalytic synthesis of adipic acid coupled with H_2_ production enhanced by a ligand modification strategy

**DOI:** 10.1038/s41467-022-32769-0

**Published:** 2022-08-25

**Authors:** Zhenhua Li, Xiaofan Li, Hua Zhou, Yan Xu, Si-Min Xu, Yue Ren, Yifan Yan, Jiangrong Yang, Kaiyue Ji, Li Li, Ming Xu, Mingfei Shao, Xianggui Kong, Xiaoming Sun, Haohong Duan

**Affiliations:** 1 State Key Laboratory of Chemical Resource Engineering, College of Chemistry, Beijing University of Chemical Technology, Beijing, China; 2 Department of Chemistry, Tsinghua University, Beijing, China; 3 Haihe Laboratory of Sustainable Chemical Transformations, Tianjin, China; 4 College of energy storage technology, Shandong University of Science and Technology, Qingdao, China; 5 College of New Materials and Chemical Engineering, Beijing Institute of Petrochemical Technology, Beijing, China

**Keywords:** Hydrogen energy, Synthesis and processing, Electrocatalysis

## Abstract

Adipic acid is an important building block of polymers, and is commercially produced by thermo-catalytic oxidation of ketone-alcohol oil (a mixture of cyclohexanol and cyclohexanone). However, this process heavily relies on the use of corrosive nitric acid while releases nitrous oxide as a potent greenhouse gas. Herein, we report an electrocatalytic strategy for the oxidation of cyclohexanone to adipic acid coupled with H_2_ production over a nickel hydroxide (Ni(OH)_2_) catalyst modified with sodium dodecyl sulfonate (SDS). The intercalated SDS facilitates the enrichment of immiscible cyclohexanone in aqueous medium, thus achieving 3.6-fold greater productivity of adipic acid and higher faradaic efficiency (FE) compared with pure Ni(OH)_2_ (93% versus 56%). This strategy is demonstrated effective for a variety of immiscible aldehydes and ketones in aqueous solution. Furthermore, we design a realistic two-electrode flow electrolyzer for electrooxidation of cyclohexanone coupling with H_2_ production, attaining adipic acid productivity of 4.7 mmol coupled with H_2_ productivity of 8.0 L at 0.8 A (corresponding to 30 mA cm^−2^) in 24 h.

## Abstract (web summary)

Adipic acid is an important building block of polymers, although its production relies on harmful reagents. Here, authors examined surfactant-modified nickel hydroxide for adipic acid electrosynthesis coupled with hydrogen gas evolution.

## Introduction

**Fig. 1:**
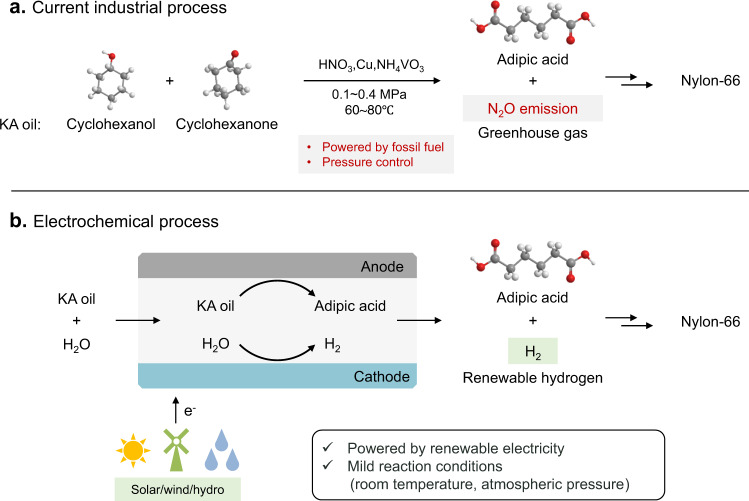
Schematic illustration of the KA oil oxidation to produce adipic acid. **a** Current industrial route. **b** Proposed electrochemical route.

Adipic acid is an important building block of polyamides and polyesters, with broad use in nylon-66, poly(butyleneadipate-co-terephthalate) and lubricant productions on an annual scale of multimillions of metric tons^1–3^. The current industrial adipic acid synthesis relies on the oxidation of ketone-alcohol (KA) oil by using 50–60% nitric acid as a strong oxidant (Fig. 1a), which requires special equipment owing to the acid corrosiveness. This process also inevitably produces stoichiometric amounts of nitrous oxide (N_2_O) as a potent greenhouse gas, with the atmospheric heat-trapping capacity nearly 300 times higher than CO_2_, thus exhaust gas processing device is necessary^4–6^. Therefore, the development of alternative pathway for adipic acid production towards a more sustainable chemical industry is highly desirable.

Electrocatalytic oxidation can offer an attractive approach for converting KA oil to adipic acid in an aqueous electrolyte under mild conditions (Fig. 1b)^7,8^. In 2004, Lyalin and Petrosyan reported a nickel oxyhydroxide (NiOOH) catalyst to achieve the electrocatalytic oxidation of cyclohexanol/cyclohexanone to adipic acid with yield of 52% at a constant current of 6 mA cm^–2^ (ref. 7). Our recent work shows the preliminary results of electrooxidation of KA oil to adipic acid with yield of 64.2% using manganese-doped cobalt oxyhydroxide catalyst^8^. Although progress made in developing electrocatalytic strategy, the corresponding research is still in its infancy with low reaction rate and low yield (<70%) of adipic acid that limit the application. One critical restriction comes from the low solubility of cyclohexanone in aqueous electrolyte, causing difficulties in mass transfer and adsorption on the catalyst surface that largely hinder reaction occurring.

For electrocatalytic conversion of molecules immiscible in aqueous electrolyte, particularly for gaseous CO_2_ and N_2_, previous literatures show that constructing a hydrophobic surface on the electrode by ligand modification is beneficial to their enrichment and therefore facilitates the reactivity^9–13^. For example, Yang and co-workers recently reported the construction of nanoparticle/ordered-ligand interlayer that contains a multi-component catalytic pocket for CO_2_ enrichment and its selective electroreduction to CO^9^. Similar strategy has also been deployed in electrode design for wastewater treatment^14,15^. The modification of sodium dodecyl benzene sulfonate on PbO_2_ anode could promote the electrochemical degradation of organic pollutants (e.g., nitrobenzene) in water^14^. Inspired by these works, we anticipate that regulating the surface hydrophobicity may enrich cyclohexanone over the catalyst surface with promoted adipic acid productivity. However, the ligand modification strategy has rarely been explored in electrocatalytic conversion of liquid molecules immiscible in aqueous medium.

In this work, we report an electrocatalytic strategy for the oxidation of cyclohexanone to adipic acid coupled with H_2_ production under mild conditions, over a nickel hydroxide catalyst modified with SDSin the interlayer (Ni(OH)_2_-SDS). The SDS modification endows the catalyst with 3.6-fold greater productivity of adipic acid than pure Ni(OH)_2_, achieving FE of 93%, representing an advantageous performance compared with the reported works. Combined experimental and molecular dynamic simulations results indicate that SDS facilitates the enrichment of cyclohexanone molecules at the edge of Ni(OH)_2_-SDS for the enhanced catalytic activity. The ligand-modified catalyst exhibits enhanced electrooxidation activity towards a broad selection of aldehydes and ketones that are immiscible in aqueous medium. Finally, we set up a realistic two-electrode membrane-free flow electrolyzer for electrooxidation of cyclohexanone coupling with H_2_ production, attaining adipic acid productivity of 4.7 mmol coupled with H_2_ productivity of 8.0 L at a constant current of 0.8 A (corresponding to 30 mA cm^−2^) in 24 h, demonstrating the potential for practical applications.

## Results

### Synthesis and structural characterizations

In previous electrocatalytic works, Ni(OH)_2_ showed activity for electrochemical oxidation of organic compounds (e.g., ethanol, glycerol, 5-hydroxymethylfurfural) in aqueous medium^16–18^. Besides, SDS is a typical surfactant with hydrophilic SO_4_^2−^ end and hydrophobic alkyl chain that can increase the affinity to water-immiscible compounds. We thus sought to prepare Ni(OH)_2_ and modify the surface with SDS, aiming at enriching cyclohexanone on the local environment of catalyst with promoted electrooxidation activity. However, SDS is soluble in water, hence the stabilization of the SDS on Ni(OH)_2_ surface remains problematic. Note that *α*-Ni(OH)_2_ is a typical two-dimensional layered material that is composed of positively-charged hydrotalcite-like host layers {[Ni(OH)_2-*x*_(H_2_O)_*y*_]^*x*+^}, and negatively-charged and adjustable interlayer anions (e.g., CO_3_^2−^, NO_3_^−^, dodecyl sulfate)^19–21^. We expect intercalation of SDS into the interlayer of *α*-Ni(OH)_2_ would prevent the dissolution of SDS into aqueous electrolyte, thus enhancing its stability.

**Fig. 2:**
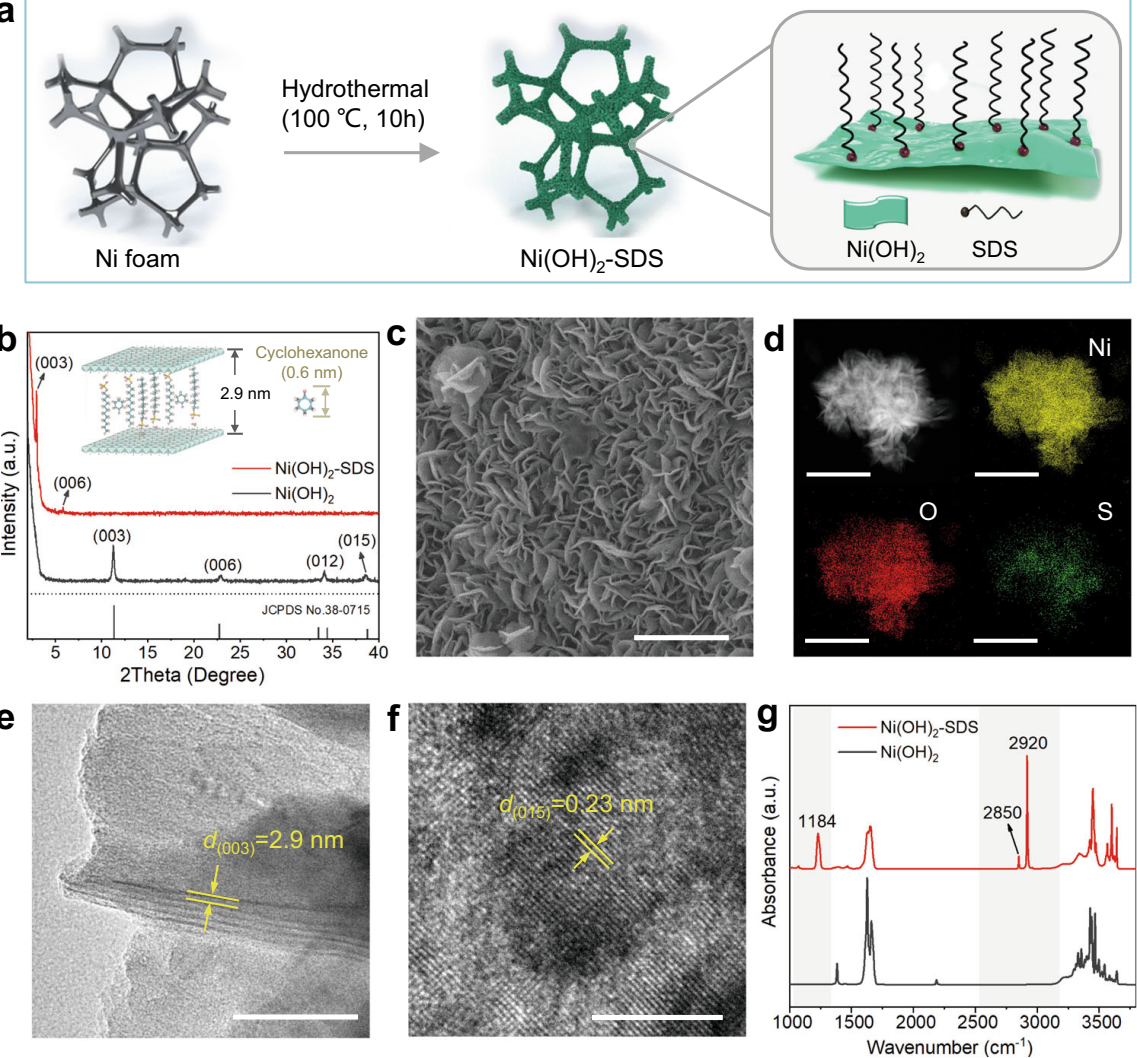
Structural characterization of the Ni(OH)_2_-SDS catalyst. **a** Schematic illustration of the synthesis of Ni(OH)_2_-SDS on Ni foam. **b**, XRD patterns of the Ni(OH)_2_-SDS and pure Ni(OH)_2_ samples. **c** SEM image of Ni(OH)_2_-SDS. Scale bar, 2 μm. **d** HAADF-STEM image of Ni(OH)_2_-SDS and corresponding element maps showing the distribution of Ni (yellow), O (red) and S (green). Scale bar, 1 μm. **e**, **f** HR-TEM images of Ni(OH)_2_-SDS. Scale bar, 50 nm **e**; 5 nm **f**. **g** FTIR spectra of the Ni(OH)_2_-SDS and pure Ni(OH)_2_.

With this idea in mind, we synthesized a SDS-modified *α*-Ni(OH)_2_ catalyst (denoted as Ni(OH)_2_-SDS) on Ni foam substrate via a hydrothermal method using Ni(NO_3_)_2_ and SDS as the precursors, by which SDS is intercalated and stabilized in the interlayer of Ni(OH)_2_ (Fig. 2a). Pure Ni(OH)_2_ was also synthesized by using a similar method without the addition of SDS. Figure 2b and Supplementary Fig. 1 shows the X-ray diffraction (XRD) patterns of as-prepared samples. The XRD pattern of pure *α*-Ni(OH)_2_ shows four diffraction peaks at 11.3°, 22.9°, 34.2° and 38.7°, which are well-indexed to the (003), (006), (012) and (015) diffraction planes of rhombohedral *α*-Ni(OH)_2_ (JCPDS No. 38-0715)^19^. For Ni(OH)_2_-SDS, the (003) diffraction peaks shift to lower degree at 2.99°, suggesting that the corresponding interlayer distance was calculated to be approximately 2.95 nm, in consistent with previous reports^21^, indicating the successful intercalating of SDS in the interlayer of Ni(OH)_2_ (inset in Fig. 2b). The scanning electron microscopy (SEM) image in Fig. 2c shows that the Ni(OH)_2_-SDS exhibits typical nanosheet array structure with thickness of ∼10 nm and diameter of 200–300 nm. The nanosheets of Ni(OH)_2_-SDS became crinkle after SDS modification in comparison with pure Ni(OH)_2_ (Supplementary Fig. 2a), which may be induced by the expansion of interlayer spacing by SDS intercalation^22,23^. Scanning transmission electron microscopy−energy-dispersive X-ray spectroscopy (STEM-EDS) mapping analysis (Fig. 2d) shows the homogeneous distributions of Ni and O throughout the nanosheet, as well as the high dispersion of S. High-resolution transmission electron microscope (HRTEM) image shows that the Ni(OH)_2_-SDS nanosheets exhibit expanded (003) plane with average interlayer spacing of ~2.9 nm (Fig. 2e), matching with the XRD results. Moreover, the HRTEM images of both Ni(OH)_2_-SDS and pure Ni(OH)_2_ display typical (015) plane with similar interplanar spacing of 0.23 nm (Fig. 2f and Supplementary Fig. 2b), suggesting that the planar structure of Ni(OH)_2_ is not obviously affected by SDS intercalation.

The presence of SDS in Ni(OH)_2_-SDS sample was verified by Fourier transform infrared spectroscopy (FTIR). As shown in Fig. 2g, the FTIR spectrum of Ni(OH)_2_-SDS shows absorption bands at 2920, 2850, and 1184 cm^–1^, corresponding to the methylene group and the sulfate head group presented in anionic SDS, respectively^22^. X-ray photoelectron spectroscopy (XPS) results show that S exists in Ni(OH)_2_-SDS, while it is absent in pure Ni(OH)_2_ (Supplementary Fig. 3). The contact angle measurements show that Ni(OH)_2_-SDS displays hydrophobic nature after SDS modification (Supplementary Fig. 4). The percentage of SDS-coordinated Ni ion sites (corresponding to the molar ratio of SDS to Ni on one side of Ni(OH)_2_ layer) was estimated to be 1:23.3 by using inductively coupled plasma-optical emission spectroscopy (ICP-OES; Supplementary Table 1, Supplementary Fig. 5, Supplementary Note 1).

### Electrocatalytic oxidation of cyclohexanone

**Fig. 3:**
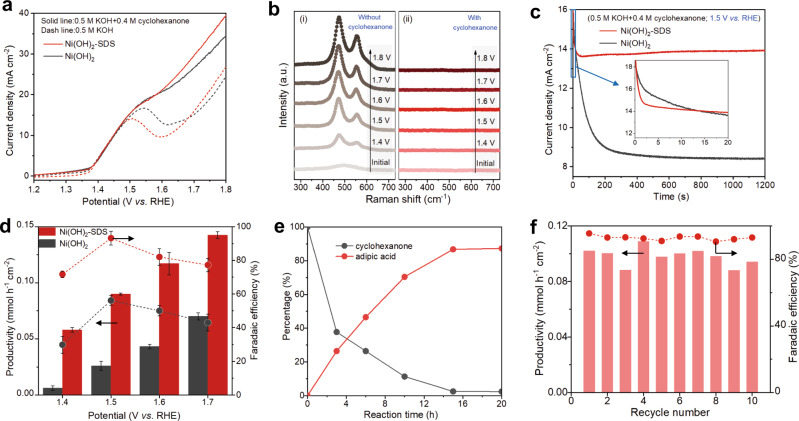
Cyclohexanone elelctrooxidation over Ni(OH)_2_ and Ni(OH)_2_-SDS catalysts. **a** LSV curves of Ni foam supported Ni(OH)_2_-SDS and pure Ni(OH)_2_ catalysts at scan rate of 10 mV s^−1^. **b** In situ Raman spectra of Ni(OH)_2_-SDS in 0.5 M KOH with or without cyclohexanone. a.u.: arbitrary units. **c** *I*-*t* curves of Ni(OH)_2_-SDS and pure Ni(OH)_2_. **d** Adipic acid productivity and FE over Ni(OH)_2_-SDS and pure Ni(OH)_2_ at different potentials. The error bars represent the standard deviation of three independent measurements by using the same sample, which is within 5%. The data represent the average value. **e** Kinetic curves for cyclohexanone electrooxidation over Ni(OH)_2_-SDS in 0.5 M KOH with 20 mM cyclohexanone at 1.5 V vs RHE. **f** Stability test of Ni(OH)_2_-SDS catalyst for cyclohexanone electrooxidation in batch reaction (10 batches; 20 h). The stirring rate of electrolyte was 800 rpm for all electrochemical tests.

We then initiated the catalytic study of electrooxidation of cyclohexanone over Ni(OH)_2_-SDS. Considering that cyclohexanone (the component of KA oil) is immiscible in water and base (Supplementary Table 2), we expect the interlayer modification of Ni(OH)_2_ by SDS with hydrophobic property may promote the catalytic activity. Pure Ni(OH)_2_ was used for comparison. Figure 3a shows the linear sweep voltammetry (LSV) curves in 0.5 M KOH solution with or without cyclohexanone. Both Ni(OH)_2_-SDS (dotted line in red) and Ni(OH)_2_ (dotted line in black) exhibit obvious oxidation peak from 1.35 to 1.6 V vs reversible hydrogen electrode (RHE), which is attributed to the reversible oxidation of Ni(OH)_2_ from Ni^2+^-OH to Ni^3+^-OOH. The two samples show comparable anodic current at potential higher than 1.6 V vs RHE that is assigned to water oxidation. After 0.4 M cyclohexanone was introduced into the KOH electrolyte, both Ni(OH)_2_-SDS and Ni(OH)_2_ exhibit higher current densities than that in pure KOH, and the onset potentials shift negatively to ∼1.4 V vs RHE, suggesting the electrooxidation of cyclohexanone is more preferable than oxygen evolution reaction (OER).

We observed that the current increasement for both Ni(OH)_2_-SDS and Ni(OH)_2_ are in line with the oxidation of Ni^2+^-OH to Ni^3+^-OOH, suggesting the formation of Ni^3+^-OOH presumably plays an important role as active phase for the electrooxidation of cyclohexanone. To confirm this point, in situ Raman spectroscopy was performed over Ni(OH)_2_-SDS. In the absence of cyclohexanone (a 0.5 M KOH solution, Fig. 3b(i)), two peaks at 473 and 553 cm^−1^ were observed from 1.35 V vs RHE, which are corresponding to the bending and stretching vibrations of NiOOH species^16,24,25^. While in the presence of cyclohexanone (Fig. 3b(ii)), the formation of NiOOH was only observed after 1.8 V vs RHE (Supplementary Fig. 6), which might be due to the spontaneous reduction of the formed NiOOH species by cyclohexanone^17,26^, as was further demonstrated by periodic sampling experiments (Supplementary Fig. 7).

We then carried out chronoamperometric (CA) measurement to investigate the cyclohexanone oxidation performance of Ni(OH)_2_-SDS (under the conditions of 0.5 M KOH with 0.4 M cyclohexanone at 1.5 V vs RHE). Intriguingly, the I-t curve (Fig. 3c) of Ni(OH)_2_-SDS exhibits more stable current density compared with pure Ni(OH)_2_ as time extends, although both samples exhibit similar initial current density. We speculate that the rapid current density decrease over pure Ni(OH)_2_ can be ascribed to the consumption of cyclohexanone on catalyst surface at the initial stage without sufficient supply from bulk solution to the catalyst surface, resulting in a diffusion-controlled process^27^. In contrast, the interclated SDS in Ni(OH)_2_ may facilitates the enrichment of cyclohexanone, leading to more stable current density. More analysis is discussed below.

### Reaction products analysis

The reaction products were quantified by high performance liquid chromatography (HPLC). As shown in Fig. 3d and Supplementary Fig. 8, the Ni(OH)_2_-SDS exhibits 3.6-fold greater productivity of adipic acid than pure Ni(OH)_2_, reaching 90 µmol cm^−2^ h^−1^ at 1.5 V vs RHE in 1 h. The FE of adipic acid over Ni(OH)_2_-SDS reaches 93% at 1.5 V vs RHE (Fig. 3d), indicating electrooxidation of cyclohexanone to adipic acid is thermodynamically more favourable and kinetically faster than other processes over Ni(OH)_2_-SDS. This can be rationalized by the lower standard potential of cyclohexanone oxidation than that of water oxidation (1.152 vs 1.23 V; Supplementary Table 3 and Supplementary Note 2). In comparison, FE of adipic acid only reaches 56% over pure Ni(OH)_2_ due to competition with OER (Supplementary Fig. 9). The reproducibility of the catalyst was also demonstrated (Supplementary Fig. 10 and Supplementary Table 4). The decrease of FE to adipic acid over Ni(OH)_2_-SDS was observed at higher potential (Fig. 3d), which is due to OER and other pathways in cyclohexanone oxidation (Supplementary Fig. 8e).

We then investigated the electrooxidation of cyclohexanone under high conversion, which is another important metric for evaluating catalysis for practical applications. The Ni(OH)_2_-SDS exhibits higher adipic acid productivity than that of pure Ni(OH)_2_ (Supplementary Fig. 11). The conversion of cyclohexanone over Ni(OH)_2_-SDS was almost completed in 16 h, and the yield of adipic acid reached 84% (Fig. 3e), representing the best catalytic performance among the reported electrocatalytic works (Supplementary Table 5).

### Catalyst stability

The stability of the Ni(OH)_2_-SDS catalyst in a long-term reaction was investigated. As shown in Fig. 3f, the productivity and selectivity of adipic acid remain relatively stable after 10 batches (overall 20 h). The maintaining of the catalyst morphology (Supplementary Fig. 12a) and the intercalated SDS (Supplementary Fig. 12b) suggest the stability of the catalyst. We investigated the number of active Ni sites of Ni(OH)_2_-SDS after 20 h of electrolysis by using pulsed CA measurement^28^, which can estimate the number of active sites accurately (Supplementary Fig. 12c). The results show that the sample exhibits almost the same number of active Ni sites as the fresh one, demonstrating the stability of the catalyst. The leaching ratio of SDS was insignificant (~0.8%) after the catalytic reaction (Supplementary Table 6), indicating that only a small amount of SDS was detached from the Ni sites after the reaction (see Supplementary Note 3 for more discussions). We ascribe the detached SDS to the ones initially stabilized on Ni sites which were reconstructed to NiOOH. Considering NiOOH phase is mostly located at the edge^29–32^, thus only representing a small proportion of the total Ni sites. Therefore, the majority of Ni(OH)_2_ structure is maintained, and the SDS molecules can be stabilized in the interlayer (Supplementary Fig. 13). In addition, the detached of SDS would not seriously affect the promoted catalytic performance, as evaluated by the contribution of SDS in the interlayer or at outer surface to the catalytic activity (Supplementary Fig. 14 and Supplementary Note 4).

### Effect of SDS in enriching cyclohexanone

Note that the electrooxidation of cyclohexanone is greatly enhanced by modification of SDS in Ni(OH)_2_, we directed our attention to understand the underlying mechanism. Several possibilities should be considered after intercalating SDS: (1) enhancement of inherent activity, (2) exposure of more active sites, (3) facilitating cyclohexanone transfer and enrichment.

Regarding scenario (1), in situ Raman spectra (Supplementary Fig. 15) show the phase transformation ability from Ni(OH)_2_ to NiOOH (the active phase) was not obviously affected by SDS intercalation. The reason may be due to the active sites are mainly at the edge of the nanosheets^29–32^, which would be less affected by the presence of SDS in the interlayer. The coordination and electronic structure of Ni(OH)_2_ were also shown less affected, as demonstrated by extended X-ray absorption fine structure spectroscopy (EXAFS; Supplementary Fig. 16 and Supplementary Table 7). Together with the aforementioned similar onset potential of the oxidation peak in the LSV curves that corresponds to Ni(OH)_2_-to-NiOOH transformation (Fig. 3a), these results imply that the two samples exhibit comparable activity for Ni(OH)_2_ reconstruction, thus the promoted activity after intercalating SDS was not due to inherent activity enhancement.

Regarding scenario (2), we measured the electrochemical active surface area (ECSA) over different samples, and Ni(OH)_2_-SDS exhibits higher ECSA than Ni(OH)_2_. We notice the ECSA value is positively correlated with the mass loading, and the latter was varied between samples from different batches (Supplementary Table 8). Therefore, the reversible sites of Ni should be taken into consideration. The LSV curves (Fig. 3a) show that the oxidation peak (Ni^2+^ to Ni^3+^) in Ni(OH)_2_-SDS is less intense than that of pure Ni(OH)_2_ in 0.5 M KOH, revealing that the modification of Ni(OH)_2_ by SDS may even hinder the exposure of Ni active sites. To compare the numbers of active sites more accurately, pulsed CA measurements were performed (Supplementary Fig. 17 and Supplementary Table 9). The pure Ni(OH)_2_ exhibits more reducible Ni sites than Ni(OH)_2_-SDS (0.443 vs 0.335 C at 0.9 V vs RHE). Therefore, exposing more active sites by SDS intercalation can be excluded. It should be noted that after normalizing the productivity of adipic acid by the numbers of reducible Ni sites, the Ni(OH)_2_-SDS exhibits productivity of 269 µmol cm^−2 ^h^−1 ^C^−1^, which is 4.8-fold higher than that of Ni(OH)_2_ (56 µmol cm^−2^ h^−1^ C^−1^) (Supplementary Table 9), confirming the promoting effect of SDS for electrooxidation cyclohexanone.

**Fig. 4:**
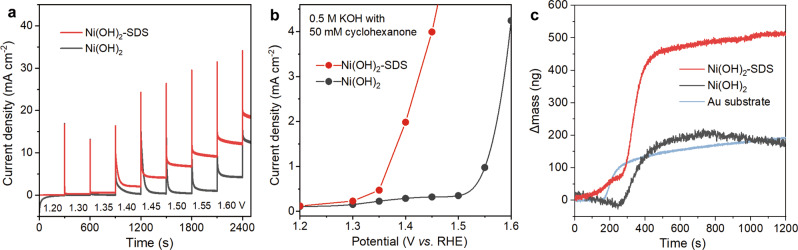
Mechanistic investigation of cyclohexanone transport and enrichment. **a** *I*–*t* curves of Ni(OH)_2_-SDS and Ni(OH)_2_ at different potentials (V vs RHE) in normal pulse voltammetry. **b** The corresponding normal pulse voltammograms of Ni(OH)_2_-SDS and Ni(OH)_2_. The measurements were conducted in 0.5 M KOH with 50 mM cyclohexanone. The current was recorded at 300 s after each potential pulse that is increasing in potential amplitude from 1.2 to 1.6 V vs RHE. **c** QCM mass response over Ni(OH)_2_-SDS, pure Ni(OH)_2_, and Au substrate in 0.5 M KOH before and after adding 0.4 M cyclohexanone.

Regarding scenario (3), to investigate if the presence of SDS in Ni(OH)_2_ interlayer would facilitate cyclohexanone transfer, normal pulse voltammetry (NPV) technique^33^ was performed in electrooxidation of cyclohexanone (Fig. 4a). The steady-state current densities at different potentials can be derived accordingly (Fig. 4b). Over pure Ni(OH)_2_, the current density was increased gradually with increasing potential from 1.2 to 1.4 V vs RHE, and then reached a plateau at potential >1.4 V vs RHE. These results suggest that mass transfer of cyclohexanone becomes more sluggish than electrooxidation at increased potential, thus giving rise to the complete consumption of cyclohexanone on electrode surface before the arrival of more cyclohexanone. Namely, the cyclohexanone concentration on electrode surface would decrease to zero at potential >1.4 V vs RHE, and mass transfer becomes the rate-determining step (RDS) in the overall process of cyclohexanone electrooxidation. In contrast, over Ni(OH)_2_-SDS, the steady-state current density was obviously higher, and was increased continuously without reaching a plateau, suggesting that the mass transfer of cyclohexanone is enhanced over Ni(OH)_2_-SDS, which is well consistent with the aforementioned stable current in CA measurements (Fig. 3c). More theoretical analysis for the facilitated mass transferof cyclohexanone by SDS intercalation is shown later.

To investigate the enrichment of cyclohexanone over Ni(OH)_2_-SDS, quartz crystal microbalance (QCM) was performed (Supplementary Fig. 18), which can reflect the ability of molecule adsorption^34^. As shown in Fig. 4c, the Ni(OH)_2_-SDS sample exhibits a significant increase in mass after 0.4 M cyclohexanone was added, which is 2.5-fold higher than that of pure Ni(OH)_2_, revealing that Ni(OH)_2_-SDS can promote the adsorption of cyclohexanone. Note that cyclohexanone adsorption was also observed on pure Ni(OH)_2_ in QCM test, which is largely contributed by the adsorption over Au substrate (pale blue curve in Fig. 4c), considering that the Au substrate cannot be completely covered by the Ni(OH)_2_ catalyst. The enrichment of cyclohexanone over Ni(OH)_2_-SDS was also demonstrated by FTIR (Supplementary Fig. 19), and by the faster transformation rate from NiOOH to Ni(OH)_2_ after introducing cyclohexanone (Supplementary Fig. 20; see detailed discussion in Supplementary Note 5).

**Fig. 5:**
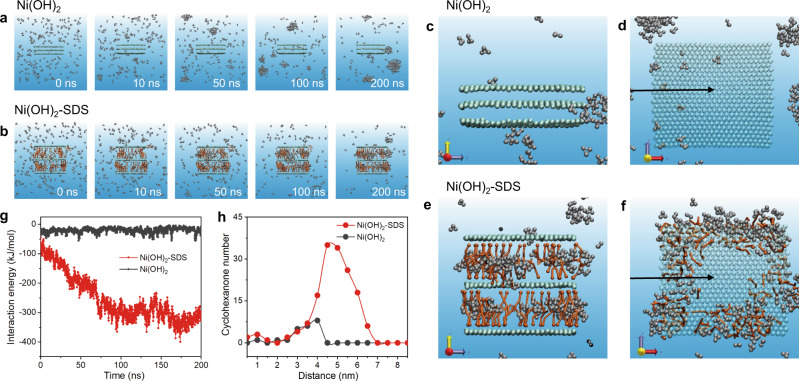
Coarse-grained molecular dynamic simulations of cyclohexanone diffusion behaviors. The time sequence of typical snapshots of cyclohexanone diffusion dynamics on **a** pure Ni(OH)_2_ and **b** Ni(OH)_2_-SDS. **c** Side and **d** top views of snapshot at 200 ns in pure Ni(OH)_2_. **e** Side and **f** top views of snapshot at 200 ns in Ni(OH)_2_-SDS. **g** Energy evolution of interaction between cyclohexanone molecules and the catalysts. The arrows in **d** and **f** show one direction for counting cyclohexanone molecules. **h** Cyclohexanone molecules distribution at different locations (four directions from bulk solution to the center of catalysts are counted). Colour codes: Ni(OH)_2_ (green), SDS (orange), cyclohexanone (silver).

To investigated whether the enriched cyclohexanone in Ni(OH)_2_-SDS has a swelling effect to the interlayer space, XRD tests over Ni(OH)_2_-SDS before and after cyclohexanone adsorption were conducted. The Ni(OH)_2_-SDS sample was immersed in a 0.5 M KOH solution with 0.4 M cyclohexanone, holding for 30 min to ensure cyclohexanone adsorption. The XRD patterns (Supplementary Fig. 21) show that the position of (003) diffraction peak that reflects the interlayer distance is almost identical after cyclohexanone adsorption, suggesting that the enriched cyclohexanone may not induce the enlargement of the interlayer space of Ni(OH)_2_-SDS. The reason can be due to cyclohexanone is mainly adsorbed at the edge or near edge of Ni(OH)_2_-SDS (Fig. 5) that would not apparently affect the inner space. In addition, the interlayer distance (2.95 nm) is much larger than the size of cyclohexanone molecule (0.6 nm). Therefore, the possible swelling effect of cyclohexanone enrichment would be weak.

### Theoretical calculations

To illuminate the mechanism of cyclohexanone diffusion from bulk electrolyte to catalyst surface, coarse-grained molecular dynamic (CGMD) simulations were carried out to provide insights into the molecular-level mechanism. The Ni(OH)_2_ model was constructed, and 124 negatively-charged SDS molecules were added randomly in the simulation box. The SDS molecules were spontaneously intercalated into the interlayer of Ni(OH)_2_ in bilayer structure due to the electrostatic attractions between Ni(OH)_2_ and head groups of SDS molecules. In order to keep the ring geometry of cyclohexanone molecules, triangle model was constructed according to rings model strategy. The complete model construction method is described in the Methods. As shown in the time sequence of typical snapshots of cyclohexanone diffusion (Fig. 5a), cyclohexanone molecules have no affinity to Ni(OH)_2_ surface, thus most of them are located in the bulk solution in random or aggregated fashions. In evident contrast, the enrichment of cyclohexanone molecules over Ni(OH)_2_-SDS is facilitated (Fig. 5b), especially at the edge and interlayer position of Ni(OH)_2_-SDS. The total time sequence of cyclohexanone diffusion in Ni(OH)_2_ (Supplementary Movie 1) and Ni(OH)_2_-SDS (Supplementary Movie 2) have been provided. The enlarged side and top views of the snapshots from 200 ns (Fig. 5c–f) show the cyclohexanone molecules were mainly aggregated at the edge of Ni(OH)_2_-SDS and fewer diffused into inner space.

In addition, the corresponding energy evolution of interaction between cyclohexanone molecules and the catalysts shows a decreased energy for Ni(OH)_2_-SDS as time extends, probably due to the hydrophobic interaction (Fig. 5g). Furthermore, the radial distribution of cyclohexanone molecules (counted along four directions from bulk solution to the center; Fig. 5h) confirm that cyclohexanone molecules tend to accumulate at the edge of Ni(OH)_2_-SDS, while no cyclohexanone was observed in the interlayer of Ni(OH)_2_. The above CGMD simulation results suggest that Ni(OH)_2_ intercalated with SDS can facilitate cyclohexanone diffusion, leading to cyclohexanone molecules accumulation mostly at the edge of the nanosheet. Considering that the active sites of Ni(OH)_2_ mainly exist at the edge of the Ni(OH)_2_ nanosheet, the oxidation of cyclohexanone can be largely enhanced. Furthermore, to investigate if the presence of more SDS in the middle of the Ni(OH)_2_ nanosheets would facilitate cyclohexanone diffusion into inner space, additional catalyst models by the introduction of more SDS molecules (275 and 578, respectively) into the simulation box were constructed. The results (Supplementary Fig. 22) show that more cyclohexanone molecules were observed into the inner space when 578 SDS molecules were intercalated, although at a much lower proportion compared with that at the edge of the nanosheet (Supplementary Fig. 22e). We ascribe these simulation results to the following reason. Once adsorbed by SDS molecules which are located at the edge of the nanosheets, cyclohexanone would be difficult to diffuse into the inner space in the limited simulation time. We anticipate that more cyclohexanone molecules would diffuse into the inner space under the real condition. In addition, more precise model structure should be established in the future work.

Furthermore, spin-polarized density functional theory (DFT) calculations were performed to understand the adsorption and release behaviors of cyclohexanone in Ni(OH)_2_-SDS. The adsorption energies of cyclohexanone in Ni(OH)_2_-SDS and solvent (water) were calculated to be ‒0.46 eV and ‒0.17 eV, respectively (Supplementary Fig. 23). As a result, the differential adsorption energy of cyclohexanone in Ni(OH)_2_-SDS and water was ‒0.29 eV, suggesting that cyclohexanone is possible to be released from Ni(OH)_2_-SDS at room temperature, especially with external disturbance under the reaction conditions, such as the strong stirring and the large concentration gradient between the interlayer and the edge of the nanosheets (see Supplementary Note 6 for more discussions). The release of the adsorbed cyclohexanones from Ni(OH)_2_-SDS was experimentally confirmed (Supplementary Fig. 24; see Supplementary Note 7 for experimental details).

### Reaction mechanism of KA oil electrooxidation

**Fig. 6:**
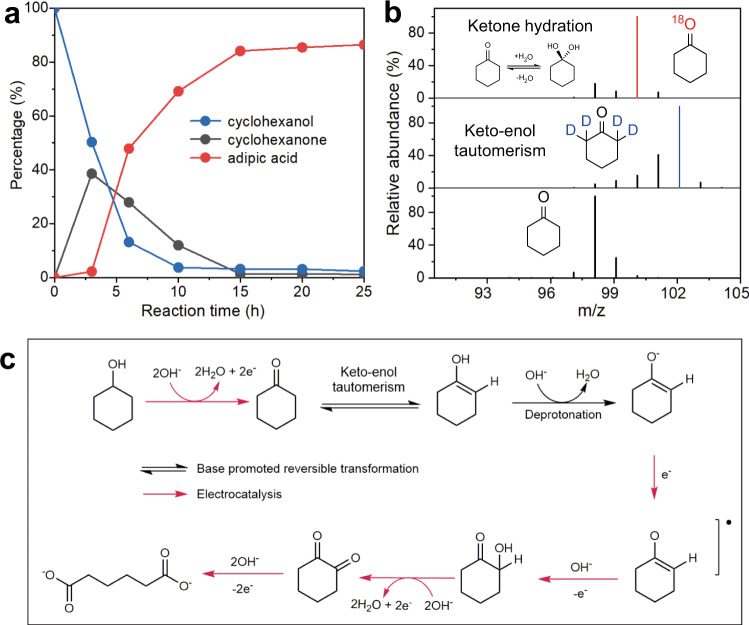
Reaction mechanism of KA oil electrooxidation. **a** Kinetic curves for cyclohexanol electrooxidation over Ni(OH)_2_-SDS in 0.5 M KOH with 20 mM cyclohexanol at 1.5 V vs RHE. **b** Mass spectra of cyclohexanone in isotope-labelled electrolyte. **c** A plausible tandem nucleophilic oxidation reaction mechanism for electrochemical oxidation of KA oil.

We then diverted our attention to cyclohexanol, which is another component of KA oil. The LSV curves in Supplementary Fig. 25a show that the electrooxidation of cyclohexanol is more favorable than that of cyclohexanone over Ni(OH)_2_-SDS, with the former achieving lower onset potential (∼1.35 V vs RHE) and higher current density (55 mA cm^−2^ at 1.7 V vs RHE) in 0.5 M KOH with 0.4 M cyclohexanol. The kinetic curves (Fig. 6a) show that cyclohexanol undergoes rapid oxidation (∼50% conversion in 3 h at 1.5 V vs RHE) to cyclohexanone and then to adipic acid, finally achieving 86.5% yield of adipic acid in 25 h. It should be noted that cyclohexanol is also immiscible in base (the solubility is slightly higher than cyclohexanone in base; Supplementary Table 2), but the promoting effect by SDS intercalation is not obvious (Supplementary Fig. 25b). We speculate that cyclohexanol can be facilely adsorbed on Ni(OH)_2_ surface with abundance hydroxyl groups via intermolecular interaction, which was demonstrated by QCM tests (Supplementary Fig. 26). In addition, adipic acid is miscible in base in the form of carboxylate, ensuring its facile release into the solution when it is generated. According to the promoted electrooxidation activity for cyclohexanone, together with the high activity for cyclohexanol, we rationalize that Ni(OH)_2_-SDS catalyst can be efficiently employed for electrooxidation of KA oil to produce adipic acid.

Based on the above results and previous literatures^7,8^, we propose a tandem nucleophilic oxidation reaction pathways for the KA oil oxidation (Fig. 6c). Cyclohexanol is firstly electrooxidized to cyclohexanone via nucleophilic dehydrogenation reaction over NiOOH, in consistent with the reported mechanism of alcohols oxidation over Ni(OH)_2_^16^. Due to the active sites are mainly located at the edge of the nanosheet, the intercalated SDS may not seriously affect the exposure of active sites (Supplementary Note 8). Then cyclohexanone is reversibly converted to enol in the presence of base. The results of isotope labeling experiments (Fig. 6b and Supplementary Fig. 27) using D_2_O and H_2_^18^O show that base promotes the reversible ketone hydration and keto-enol tautomerism to form enol or gem-diol intermediates^8,35^. According to the proposed mechanism by Lyalin and Petrosyan,^36^ cyclohexanone in enol form can be spontaneously converted to enolate anion in base and then to O-centered radical, which is further transformed to hydroxy cyclohexanone, and then cyclohexane 1,2-dione is afforded by nucleophile dehydrogenation. Finally, the formed cyclohexane 1,2-dione is oxidized to adipic acid. By using cyclohexane 1,2-dione as the substrate for electrooxidation (Supplementary Fig. 28), we obtained adipic acid as the product, suggesting the electrooxidation of cyclohexanol/cyclohexanone plausibly proceeds via cyclohexane 1,2-dione as the intermediate. Note that glutaric acid was observed over Ni(OH)_2_-SDS and Ni(OH)_2_ in the HPLC spectra at high potentials (Fig. 3d and Supplementary Fig. 8e), which was probably generated through the formation of cyclohexane 1,3-dione intermediate during cyclohexanone oxidation (Supplementary Fig. 29)^36^.

### Universality of the ligand modification strategy

**Fig. 7:**
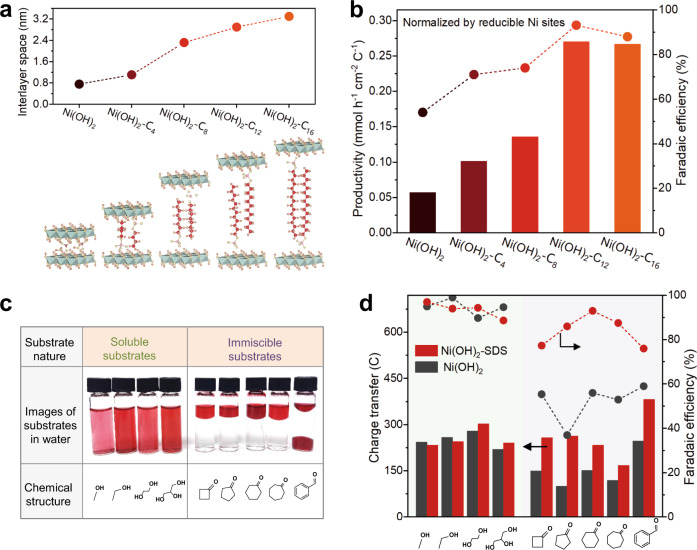
Universality of the ligand modification strategy. **a** Interlayer space of different Ni(OH)_2_-C_*n*_ samples that detected by XRD. **b** Reducible Ni sites-normalized productivity and FE of adipic acid over different Ni(OH)_2_-C_*n*_ samples in 0.5 M KOH with 0.4 M cyclohexanone at 1.5 V vs RHE (stirring rate = 800 rpm). **c** Photo of different substrates in 0.5 M KOH solution, the substrates are marked by oil-soluble pigment (Pigment Red 122). **d** The charge transfer and corresponding FE of products over Ni(OH)_2_-SDS and pure Ni(OH)_2_ in 0.5 M KOH with 0.4 M of different substrates at 1.5 V vs RHE (stirring rate = 800 rpm).

To demonstrate the universality of the ligand modification strategy for other surfactants, we prepared Ni(OH)_2_ intercalated with surfactants composing of SO_4_^−^ as the end group and alkyl chain ranging from C_4_ to C_16_. The as-prepared samples are denoted as Ni(OH)_2_-C_*n*_ (*n* represents the alkyl chain length of surfactants, C_4_, C_8_, C_12_, and C_16_ were used in the study; C_12_ corresponds to SDS). The interlayer space of Ni(OH)_2_ can be expanded from 0.76 nm (corresponds to pure Ni(OH)_2_) to 3.36 nm (corresponds to Ni(OH)_2_-C_16_) that were estimated by XRD (Fig. 7a and Supplementary Fig. 30), in consistency with the length of the ligands (Supplementary Table 10), indicating their successful intercalation into the interlayer of Ni(OH)_2_ at perpendicular adsorption configuration^37^.

The catalytic results (Supplementary Fig. 31) show that all the Ni(OH)_2_-C_*n*_ samples exhibit higher current density than pure Ni(OH)_2_ in electrooxidation of cyclohexanone (in 0.5 M KOH with 0.4 M cyclohexanone at 1.5 V vs RHE). Since the Ni(OH)_2_-C_*n*_ samples exhibit different mass loading (Supplementary Table 11) and size (Supplementary Fig. 32), which may influence the exposure of active sites and thus the electrooxidation performance, we calculated the productivity normalized by ECSA (Supplementary Table 11) and also by the numbers of reducible Ni sites via aforementioned pulsed CA measurements (Fig. 7b, Supplementary Fig. 33 and Supplementary Table 12). The results show that the productivities of Ni(OH)_2_-C_*n*_ samples were higher than that of pure Ni(OH)_2_, demonstrating the promoting effect of interlayer surfactants for electrooxidation of cyclohexanone.

To demonstrate the feasibility of the SDS-intercalated strategy for other substrates immiscible in water, we assessed a series of immiscible compounds (e.g., cyclobutanone, cyclopentanone, cycloheptanone and benzaldehyde) (Fig. 7c, right), as well as miscible ones (e.g., methanol, ethanol, ethylene glycol, and glycerol) (Fig. 7c, left) for comparison. The solubility of these substrates can be examined by introducing Pigment Red 122 as a oil-soluble pigment into the system, by which a homogeneous red solution would be formed for the miscible substrates, while transparent water phase and red oil phase would be generated for the immiscible ones (Fig. 7c). For electrooxidation of the immiscible substrates (Fig. 7d,), the Ni(OH)_2_-SDS show superior performance compared with pure Ni(OH)_2_ in terms of current density and FE to adipic acid. In constrast, for miscible substrates, there is no significant cataltyic difference between Ni(OH)_2_-SDS and pure Ni(OH)_2_ (Supplementary Fig. 34). These results indicate the efficacy of ligand modification on promoting the catalytic performance of immiscible compounds oxidation.

In addition, the ligand modification strategy is applicable to other layered materials including Co(OH)_2_, CoMn-, NiCo-, NiFe-layered double hydroxides (LDHs; Supplementary Fig. 35). After SDS modification in the interlayer, all the layered materials show enhanced productivity and FE in cyclohexanone electrooxidation to adipic acid to a similar extent. Therefore, the combined results including intercalating surfactants with different length of alkyl chain, employing various immiscible substrates, and evaluating different layered materials together suggest the universality of the ligand modification strategy in enriching immiscible substrates for enhanced electrooxidation performance.

### Membrane-free flow electrolyzer

**Fig. 8:**
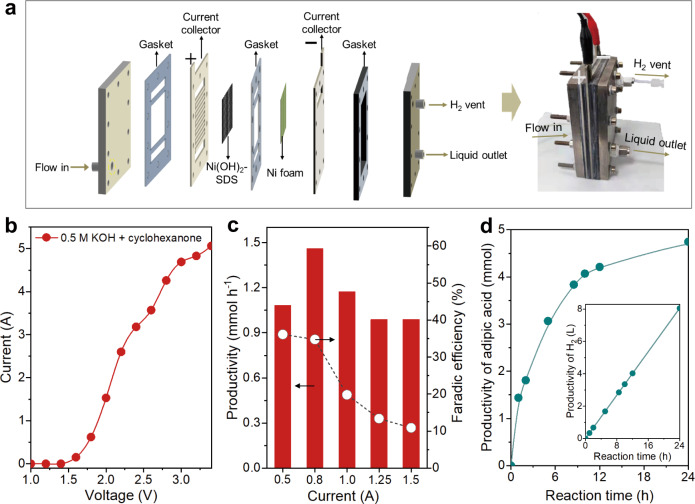
Membrane-free flow electrolyzer. **a** Schematic illustration and photograph of the membrane-free flow electrolyzer. **b** LSV curves of Ni(OH)_2_-SDS in the membrane-free flow electrolyzer. **c** Adipic acid productivity and FE at different current for 1 h. **d** Productivity of adipic acid and H_2_ (inset) in the membrane-free flow electrolyzer at 0.8 A (30 mA cm^−2^). The electrolyte for all the tests was 0.5 M KOH with 50 mM cyclohexanone.

To evaluate the catalytic performance of Ni(OH)_2_-SDS in a more practical-relevant condition, we set up a homemade membrane-free flow electrolyzer and carried out two-electrode tests, by using Ni(OH)_2_-SDS catalyst as the anode and Ni foam as the cathode with working area of 36 cm^2^ (Fig. 8a and Supplementary Fig. 36). A homogenous electrolyte (50 mM cyclohexanone in 0.5 M KOH) was circulated in the flow reactor by using a peristaltic pump at a flow rate of 3 mL min^–1^. The LSV curve in (Fig. 8b) shows that electrooxidation of cyclohexanone took place from ∼1.5 V and reach current of 4.5 A (corresponding to 125 mA cm^−2^) at 2.9 V (in 0.5 M KOH with 50 mM cyclohexanone). The current is higher than that in pure 0.5 M KOH (Supplementary Fig. 37), demonstrating cyclohexanone electrooxidation is preferable than OER.

We then performed constant current tests to evaluate the performance of adipic acid production in the electrolyzer. The results (Fig. 8c) show that the productivity of adipic acid was increased when the current was elevated from 0.5 to 0.8 A, whereas the productivity and FE were decreased with further increase of current due to the competitive OER process. We then evaluated a long-term electrolysis for 24 h at constant current of 0.8 A (30 mA cm^−2^). The results show that adipic acid was obtained with productivity of 4.7 mmol and yield of 64% (Fig. 8d and Supplementary Fig. 38). Meanwhile, cathodic H_2_ was produced at cathode reaching 8.0 L in 24 h (Fig. 8d, inset). We notice that the adipic acid formation rate is decreased with the extension of time, which is attributed to the reduced cyclohexanone concentration, considering that the electrolyte was circulated into the reactor multiple times (see more discussions in Supplementary Note 9). Indeed, the catalytic performance of Ni(OH)_2_-SDS can be restored by replenishing with fresh electrolyte with the standard deviation within 10% (Supplementary Fig. 38). The above results suggest the potential of this electrocatalytic strategy in coproduction of adipic acid and H_2_ under a more realistic conditions.

## Discussion

In industry-related applications, substrate with higher concentration is often needed. Under this scenario, how to avoid the phase separation would be a realistic challenge for immiscible compounds in aqueous electrolyte. In order to improve the solubility of the materials, mixed solvent consisting of water and organic solvent can be used, and more optimizing work awaits to be done. Moreover, for the product separation (e.g., adipic acid), we propose an electrocatalysis-coupling-electrodialysis (EC-ED) system might be helpful to separate adipic acid and simultaneously reuse the alkali, thus reducing the separation cost to a great extent (Supplementary Fig. 39 and Supplementary Note 10). However, it remains difficult to systematically evaluate the feasibility of this concept at the current stage, and we are endeavoring to carry out more studies on the separation and purification of adipic acid.

In summary, we achieved the electrooxidation of KA oil to adipic acid by using a SDS-modified Ni(OH)_2_ catalyst. By showing experimental and molecular dynamic simulations evidence, we demonstrate that the interclated SDS in Ni(OH)_2_ facilitates cyclohexanone mass transfer and enriches cyclohexanone at the edge of the nanosheets, leading to 3.6-fold higher oxidation activity toward adipic acid formation and higher FE compared with pure Ni(OH)_2_. The ligand modification strategy is applicable for immiscible aldehydes and ketones with enhanced electrooxidation activity. Furthermore, we evaluated the reaction coupling with H_2_ production in a more realistic two-electrode membrane-free flow electrolyzer, revealing high current for the potential for further practical explorations. This work offers a promising strategy to facilitate the electrocatalytic conversion of immiscible substrates for valuable chemicals production by engineering catalyst surface for strengthened substrate enrichment.

## Methods

### General information

Except noted, all chemicals were purchased and used without further purification.

### Catalyst preparation

The Ni(OH)_2_-SDS was synthesized using a hydrothermal method on the Ni foam substrate. Typically, Ni(NO_3_)_2_·6H_2_O (1.5 mmol), NH_4_F (4.16 mmol), urea (16.6 mmol), and SDS (3.0 mmol) were dissolved in 40 mL deionized water under vigorous stirring for 30 min, which was then transferred to a Teflon-lined stainless-steel autoclave. A piece of Ni foam (2 × 3 × 0.15 cm; pretreated with 0.5 M HCl, ethanol, and deionized water, each for 15 min) was introduced in the autoclave, sealed, and kept at 100 °C for 10 h. The substrate coated with Ni(OH)_2_-SDS was then withdrawn, washed thoroughly with H_2_O and dried at 60 °C for 12 h. The Ni(OH)_2_ sample was synthesized by the same method without the addition of SDS. The Ni(OH)_2_-C_*n*_ (*n* = 4, 8, 16) samples were synthesized by the same method by changing SDS with sodium butyl sulfate (Ni(OH)_2_-C_4_), sodium octyl sulfate (Ni(OH)_2_-C_8_), and sodium hexadecyl sulfate (Ni(OH)_2_-C_16_).

### Characterization

X-ray powder diffraction (XRD) patterns were recorded using a Shimadzu XRD-6000 diffractometer equipped with a graphite-filtered Cu Kα radiation source (*λ* = 0.15418 nm). X-ray photoelectron spectra (XPS) were performed on a Thermo VG ESCALAB 250 X-ray photoelectron spectrometer at a pressure of about 2 × 10^−9^ Pa using Al Kα X-rays as the excitation source. Scanning electron microscope (SEM) images were taken on a Zeiss SUPRA 55 Field Emission scanning electron microscope operated at 20 kV. Transmission electron microscopy (TEM) images were recorded with JEOL JEM-2010 high resolution (HR-) TEM operated at 200 kV, combined with energy dispersive X-ray spectroscopy (EDX). The Ni XAFS measurements was performed at the beamline 1W1B of the Beijing Synchrotron Radiation Facility (BSRF), Institute of High Energy Physics (IHEP), Chinese Academy of Sciences (CAS). Extended X-ray absorption fine structure spectra (EXAFS) were recorded at ambient temperature in transmission mode. The typical energy of the storage ring was 2.5 GeV with a maximum current of 250 mA; the Si (111) double crystal monochromator was used. Fourier transform of the EXAFS spectra were carried out in a *K*-range from 3.0 to 12.8 Å^−1^. Surface contact angle of catalysts was measured using a sessile drop at three different sites of each sample using a commercial drop shape analysis system (DSA100, KRüSS GmbH, Germany). The volume of water droplets used for measurement is 2 μL. FTIR spectra of cyclohexanone were carried out in a Bruker Equinox 55 spectrometer, between 4000 and 400 cm^−1^ with a resolution of 4 cm^−1^ after 600 scans per spectrum. Quartz crystal microbalance (QCM) analyses was conducted using a Q-Sense E4-Auto system (Paramus, NJ) at room temperature (Bioline Scientific) using Au-coated quartz sensors.

In situ Raman spectra were recorded on a confocal Raman microspectrometer (Renishaw, inVia-Reflex, 532 nm) using a 632 nm laser and the power was set at 2 mW under different potentials monitored by a CHI 760E electrochemical workstation equipped with an in situ Raman cell (Beijing Scistar Technology Co. Ltd). The applied potentials during the in situ Raman tests were not corrected by the solution resistance, which is constant with the LSV test without I-R correction (Fig. 3a), ensuring clear comparison of the results by using different techniques.

In situ FTIR spectra to investigate the adsorption of cyclohexanone on Ni(OH)_2_ and Ni(OH)_2_-SDS were recorded on a Bruker Equinox 55 spectrometer equipped with a cell fitted with BaF_2_ windows (Beijing Scistar Technology Co. Ltd) and an MCT-A detector cooled with liquid nitrogen. The spectrum was collected at a resolution of 4 cm^−1^ with an accumulation of 64 scans in the range of 4000–1000 cm^−1^. About 30 mg of the sample was pressed into a wafer with a diameter of 13 mm, which was then installed in an in situ IR cell. The sample was pre-processing by He gas at r.t. for 1 h and then cyclohexanone was flowed into the cell for 15 min, then physically adsorbed cyclohexanone was removed by flowing He gas for 15 min. The FTIR spectra were in situ collected during the He purging process.

### Electrochemical measurements

All electrochemical experiments for cyclohexanone and cyclohexanol oxidation were performed in 0.5 M KOH electrolyte at room temperature on an electrochemical workstation (CHI 760E, CH Instruments, Inc.). The electrochemical tests were performed in a membrane-free glass beaker, using Ag/AgCl electrode (with saturated KCl as the filling solution) and a platinum foil as reference and counter electrodes, respectively. All potentials mentioned in this work were converted to potentials versus RHE (in volts) according to Eq. 1:

1 $${E}_{{{{{{\rm{RHE}}}}}}}={E}_{{{{{{\rm{Ag}}}}}}/{{{{{\rm{AgCl}}}}}}}+{E}_{{{{{{\rm{Ag}}}}}}/{{{{{\rm{AgCl}}}}}}\; {{{{{\rm{vs}}}}}}\, {{{{{\rm{NHE}}}}}}}+0.059 \,{{{{{\rm{pH}}}}}}$$

where *E*_Ag/AgCl vs NHE_ in Eq. 1 is 0.197 V at 20 °C.

The FEs of the products were calculated based on their corresponding electron transfer per molecule oxidation using the following equation.

2 $${{{{{\rm{Faradaic}}}}}}\,{{{{{\rm{efficiency}}}}}}=\frac{{e}_{{{{{{\rm{products}}}}}}}\times {n}_{{{{{{\rm{products}}}}}}}{\times} {N}}{{Q}/{n}}\times 100{{{\%}}}$$

where *e*_products_ is the number of electrons required to oxidize substrates to products, *n*_products_ is the productivity of products, *N* is Avogadro’s constant (*N* = 6.02 × 10^23^), *Q* is the quantity of electric charge, and *n* is the elementary charge (*e* = 1.602 × 10^−19^ C).

The acid products were quantified by high performance liquid chromatography (HPLC; Angilent 1200 Infinity Series) equipped with organic acid column (Coregel 87H3) using 5 mM aqueous H_2_SO_4_ as mobile phase and detected by UV detector (210 nm) and refractive index detector. The reactants (cyclohexanone and cyclohexanol) were quantified by ^1^H NMR (Bruker Avance III 400 HD spectrometer).

Oxygen production was measured using a Neo-FOX oxygen-sensing system (Ocean Optics Inc.) equipped with a FOX Y probe inserted into the headspace of an airtight cell. The cell was purged with nitrogen for 2 h prior to measurement, and then the oxygen evolution was monitored at 1.5 V vs RHE.

Isotope labeling experiments were performed by adding 20 mM cyclohexanone in alkaline H_2_O, H_2_^18^O or D_2_O solvent (0.5 M KOH). After 30 min of stirring, 1 mL of solution was taken out from the cell and analyzed using gas chromatography mass spectrometry (GC-MS, Agilent Technologies, GC7890B, MS 5977B).

### Coarse-grained molecular dynamic simulations

Coarse-grained molecular dynamic (CGMD) simulations were applied in this study^38,39^. Compared with atomic simulation models, the coarse-grained models, which map a group of atoms into one single site, allow simulations to be carried out with a larger length scale and longer timescale. All simulations were done by GROMACS 4.6.7^40^ with Martini force field^41^. The Ni(OH)_2_ model was constructed by arranging a number of hydrophilic Qda-type beads into 3 layer sheet structure of 7.5 nm in side length and 0.7 nm in interlayer distance (Qda is a parameter in Martini force field). The charge of each interaction site was set to +1 so that the Ni(OH)_2_ matrix was cationic. To decorate the electrode with surfactants, 124 negatively-charged SDS molecules were added randomly in the simulation box. Four hydrophobic alkyl groups were set as a single type C1 CG bead, and the hydrophilic head group (-SO_4_^-^) of SDS was represented by a Qa CG bead with a negative charge^42^. The SDS molecules spontaneously intercalated into the interlayer of Ni(OH)_2_ in bilayer structure due to the electrostatic attractions between Ni(OH)_2_ and head groups of SDS molecules, resulting in the interlayer distance of Ni(OH)_2_ increased to 2.9 nm. In order to keep the ring geometry of cyclohexanone molecules, triangle model was constructed according to rings model strategy^41^. The initial size of simulation box was 16.0 × 16.0 × 16.0 nm^3^ and full of water molecules with account neutralizing ions. NPT ensemble (constant particle number, pressure and temperature) was used in our simulation. Temperature is kept constant at 300 K using the Berendsen weak coupling algorithm with a time constant of 2 ps. The neighbor list for non-bonded interactions was updated every 10 steps. For all simulations, a cutoff of 1.2 nm was used for van der Waals interactions. The Lennard-Jones potential is smoothly shifted to zero between 0.9 nm and 1.2 nm to reduce the cutoff noise. For electrostatic interactions, the coulombic potential, with a cutoff of 1.2 nm, is smoothly shifted to zero from 0 to 1.2 nm. Here, a time step of 20 fs was used for all simulations. Periodic boundary conditions were implemented in all three directions. Snapshots were rendered by VMD^43^.

### Spin-polarized DFT calculations

The detailed information for model construction and computational methods of the spin-polarized DFT calculations is described in the Supplementary Note 11.

## Supplementary information

Supplementary Information

Description of Additional Supplementary Files

Supplementary Movie 1

Supplementary Movie 2

## Data Availability

The data supporting the findings of this study are available within the article and its Supplementary Information. The Source data are provided with this paper and available in the figshare repository (10.6084/m9.figshare.20365536.v1). Additional data are available from the corresponding authors on reasonable request.
