# Supplementary material for: Electrocatalytic synthesis of adipic acid coupled with H2 production enhanced by a ligand modification strategy
Source: Nat Commun. 2022 Aug 25;13:5009. doi: 10.1038/s41467-022-32769-0 (PMC9411531; doi:10.1038/s41467-022-32769-0)
Supplement: Supplementary file 1 — Supplementary Information [file 41467_2022_32769_MOESM1_ESM.pdf]

Supplementary Information

Electrocatalytic Synthesis of Adipic Acid Coupled with H_2 Production Enhanced by a Ligand Modification Strategy

Zhenhua Li[1,#], Xiaofan Li[1,#], Hua Zhou[1,2,3,#], Yan Xu[4,#], Si-Min Xu[1], Yue Ren[1], Yifan Yan[1], Jiangrong Yang[1], Kaiyue Ji[2], Li Li[5], Ming Xu[1], Mingfei Shao[1], Xianggui Kong[1], Xiaoming Sun[1] and Haohong Duan[2,3]*

[1]State Key Laboratory of Chemical Resource Engineering, College of Chemistry, Beijing University of Chemical Technology, Beijing 100029, China

[2]Department of Chemistry, Tsinghua University, Beijing 100084, China

[3]Haihe Laboratory of Sustainable Chemical Transformations, Tianjin 300192, China

[4]College of energy storage technology, Shandong University of Science and Technology, Qingdao 266590, China

[5]College of New Materials and Chemical Engineering, Beijing Institute of Petrochemical Technology, Beijing 102617, China

[#]Zhenhua Li, Xiaofan Li, Hua Zhou and Yan Xu contributed equally to this work.

Correspondence and requests for materials should be addressed to H.D. (email: hhduan@mail.tsinghua.edu.cn).

Supplementary figures

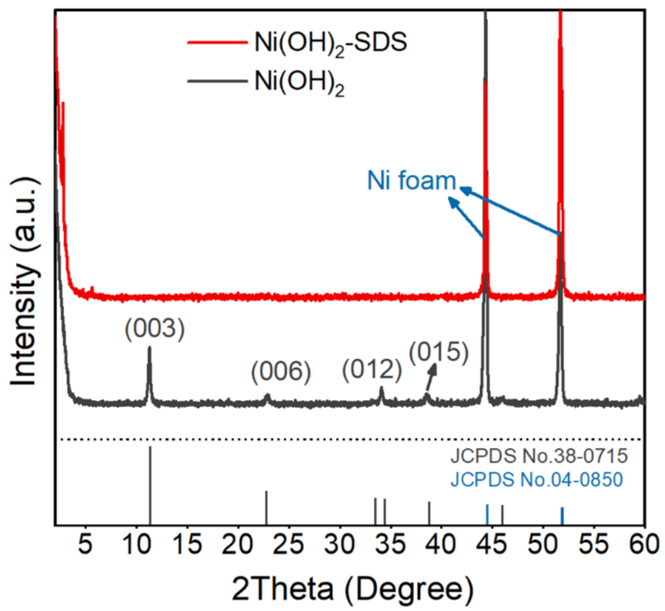

Supplementary Fig. 1 Characterizations of different catalysts. XRD patterns of the $Ni(OH)_2$-SDS and pure $Ni(OH)_2$.

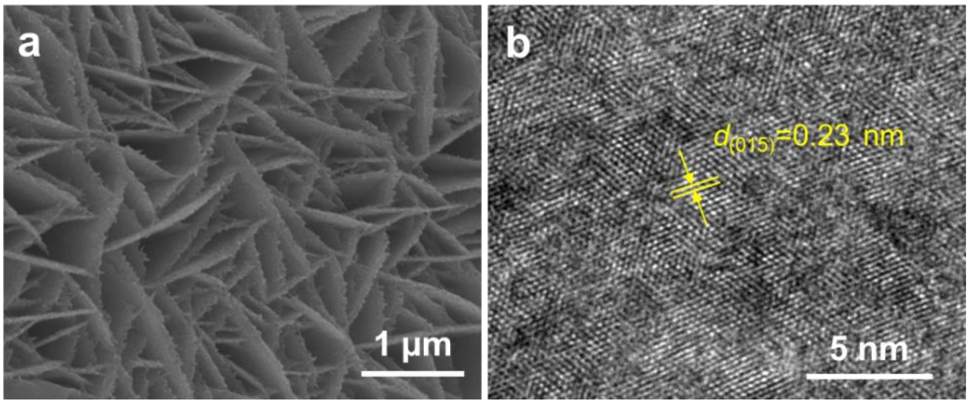

Supplementary Fig. 2 Characterizations of $Ni(OH)_2$. a, SEM image and **b**, HRTEM image of pure $Ni(OH)_2$ sample.

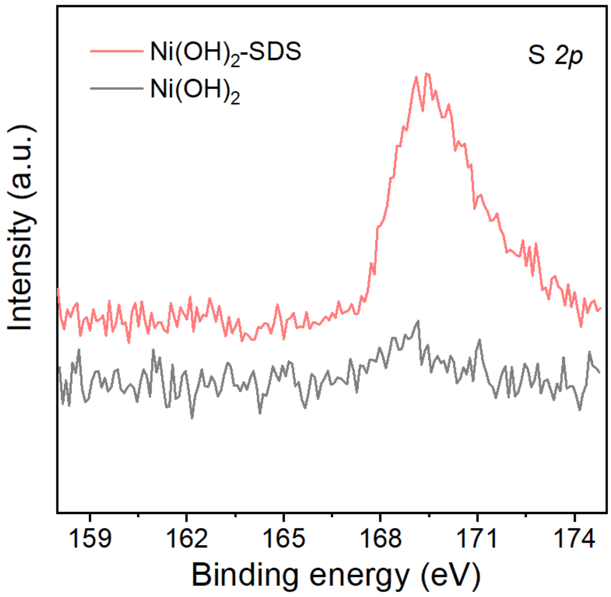

Supplementary Fig. 3 Characterizations of different catalysts. S *2p* XPS spectra of $Ni(OH)_2$-SDS and $Ni(OH)_2$.

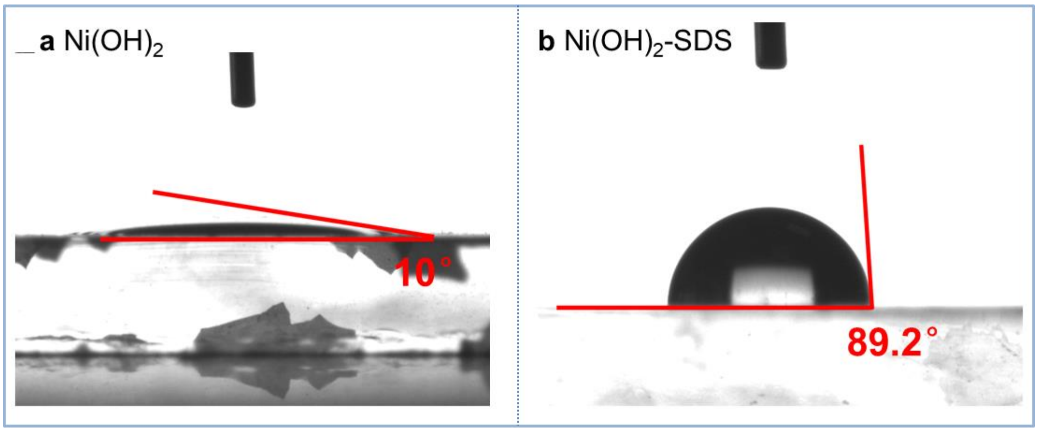

Supplementary Fig. 4 Hydrophobic performances. Surface contact Angle test results of **a**, pure $Ni(OH)_2$ and **b**, $Ni(OH)_2$-SDS.

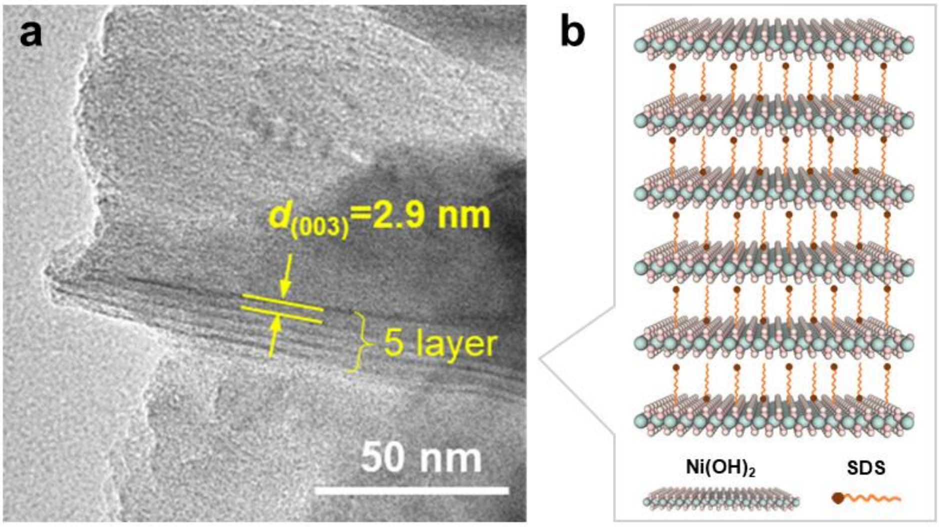

Supplementary Fig. 5 Characterization of SDS intercalation. a, HRTEM image of $Ni(OH)_2$-SDS showing the thickness of the nanosheet. **b**, the schematic structure of $Ni(OH)_2$-SDS (right). The discussion on the schematic structure and the calculation process of the percentage of SDS-coordinated Ni ion sites have been displayed as Supplementary Note 1.

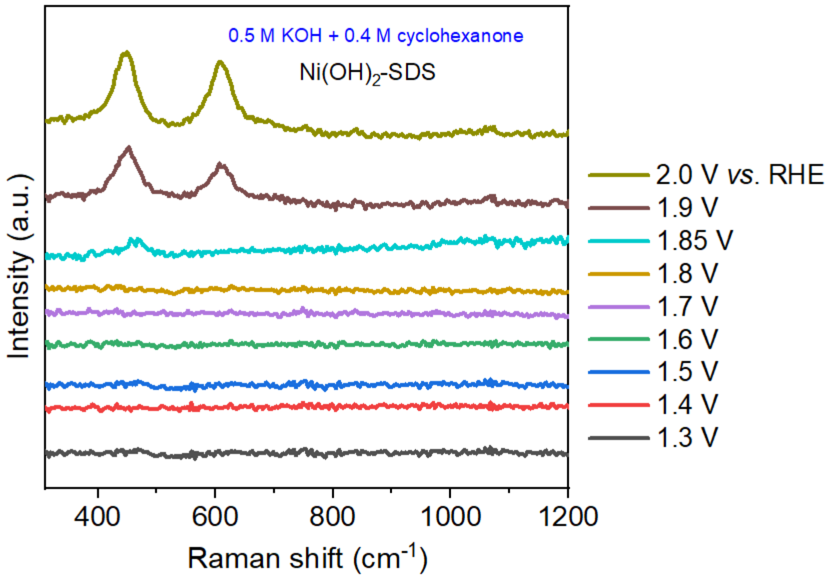

Supplementary Fig. 6 In situ Raman spectra. In situ Raman spectra of $Ni(OH)_2$-SDS in 0.5 M KOH with 0.4 M cyclohexanone.

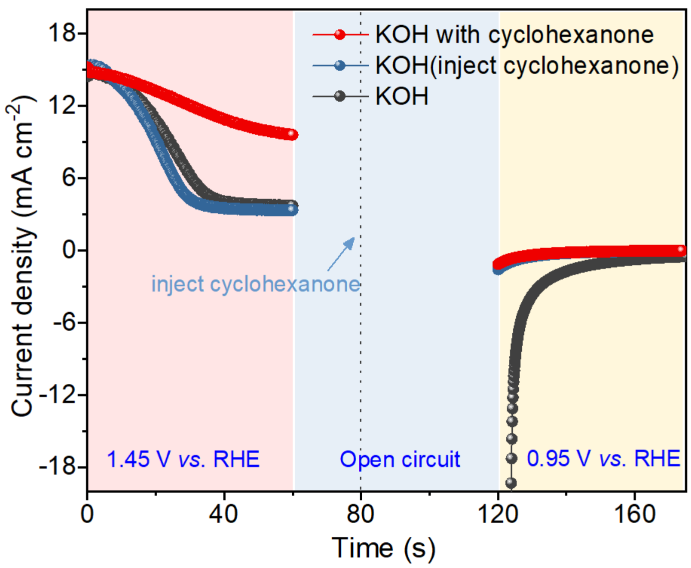

Supplementary Fig. 7 Periodic sampling experiments. Periodic sampling experiments of cyclohexanone oxidation over $Ni(OH)_2$-SDS at 1.45 V (0 to 60 s), open circuit state (60 to 120 s), and at 0.95 V (120 to 180 s. The periodic sampling experiments show that NiOOH intermediate is formed on $Ni(OH)_2$-SDS catalyst at 1.45 V vs RHE and remains stable at open circuit voltage in a 0.5 M KOH solution. After cyclohexanone was injected at the open circuit stage, NiOOH cannot be probed at a lower potential (0.95V vs RHE), indicating that electrons and protons are transferred from cyclohexanone to NiOOH spontaneously. As a result, the cyclohexanone electrooxidation over $Ni(OH)_2$ follows a two-step reaction with single electron transfer process, which involves an electrochemical dehydrogenation of $Ni(OH)_2$ to form NiOOH containing electrophilic lattice oxygen ($Ni^{2+}(OH)_2 + OH^- \rightarrow Ni^{3+}OOH + H_2O + e^-$), followed by the spontaneous nucleophile dehydrogenation reaction ($Ni^{3+}OOH + H_{Nu} + e^-_{Nu} \rightarrow Ni^{2+}(OH)_2$).

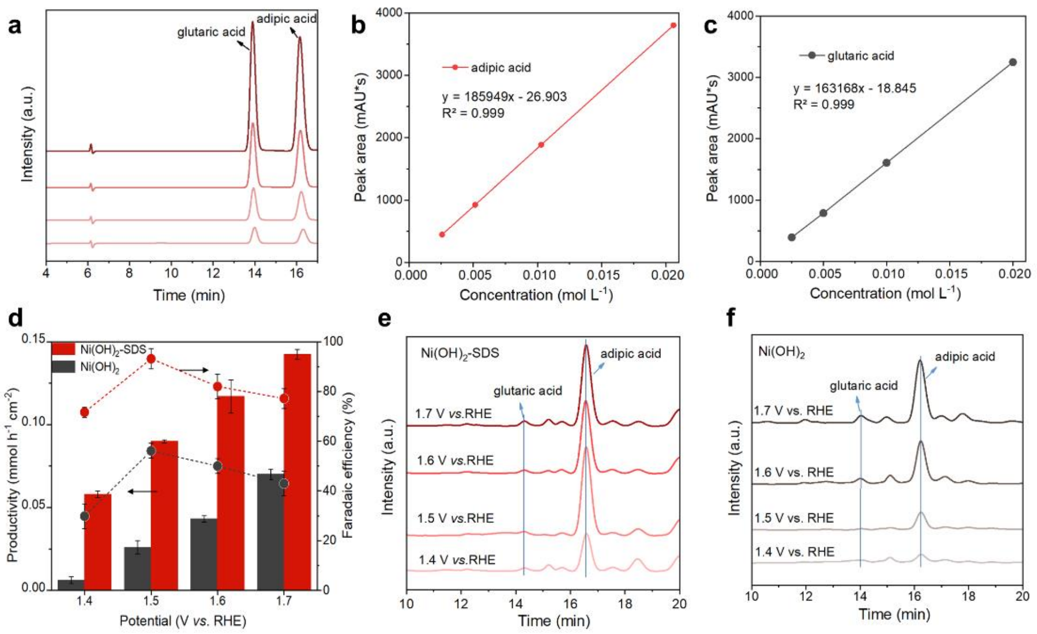

Supplementary Fig. 8 Products quantification. a, HPLC spectra of standard samples of adipic acid and glutaric acid, and the corresponding standard curves of **b**, adipic acid and **c**, glutaric acid. **d**, Adipic acid productivity and FE over $Ni(OH)_2$-SDS and pure $Ni(OH)_2$ at different potentials in 0.5 M KOH with 0.4 M cyclohexanone, and the corresponding HPLC spectra of cyclohexanone oxidation over **e**, $Ni(OH)_2$-SDS and **f**, pure $Ni(OH)_2$ at different potentials.

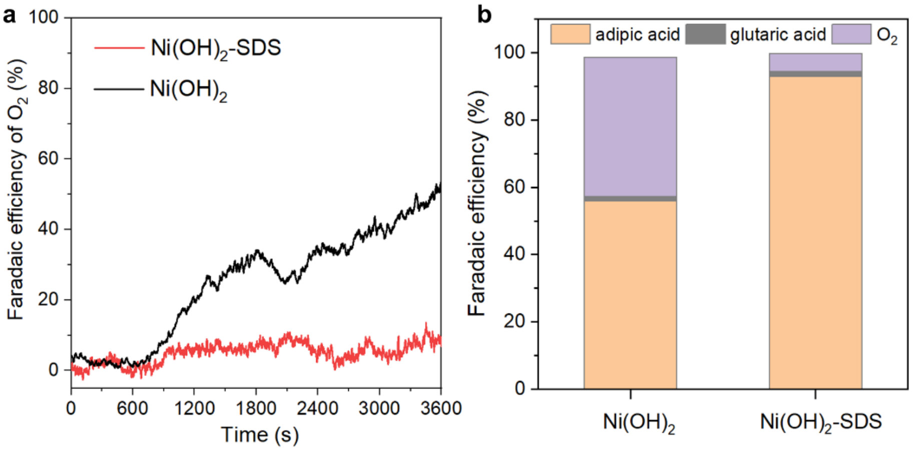

Supplementary Fig. 9 OER performances. a, FE of O_2 by OER over pure $Ni(OH)_2$ and $Ni(OH)_2$-SDS in 0.5 M KOH with 0.4 M cyclohexanone at 1.5 V vs RHE. **b**, FEs of adipic acid, O_2 and glutaric acid (the by-product of cyclohexanone oxidation) during the 1-hour test.

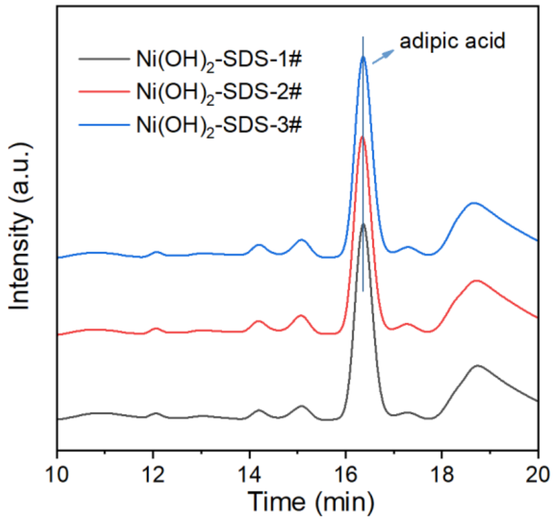

Supplementary Fig. 10 Reproducibility of the catalyst. HPLC spectra of cyclohexanone oxidation over $Ni(OH)_2$-SDS catalysts synthesized from three batches at 1.5 V vs RHE in 0.5 M KOH with 0.4 M cyclohexanone in 1 h.

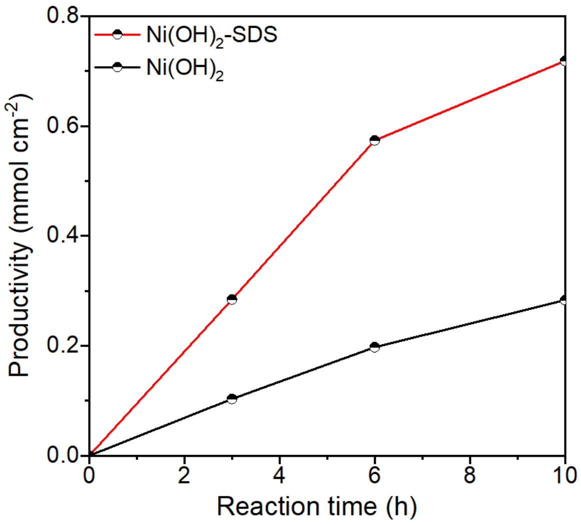

Supplementary Fig. 11 Adipic acid productivity. Adipic acid productivity over $Ni(OH)_2$ and $Ni(OH)_2$-SDS in 0.5 M KOH with 20 mM cyclohexanone at 1.5 V vs RHE.

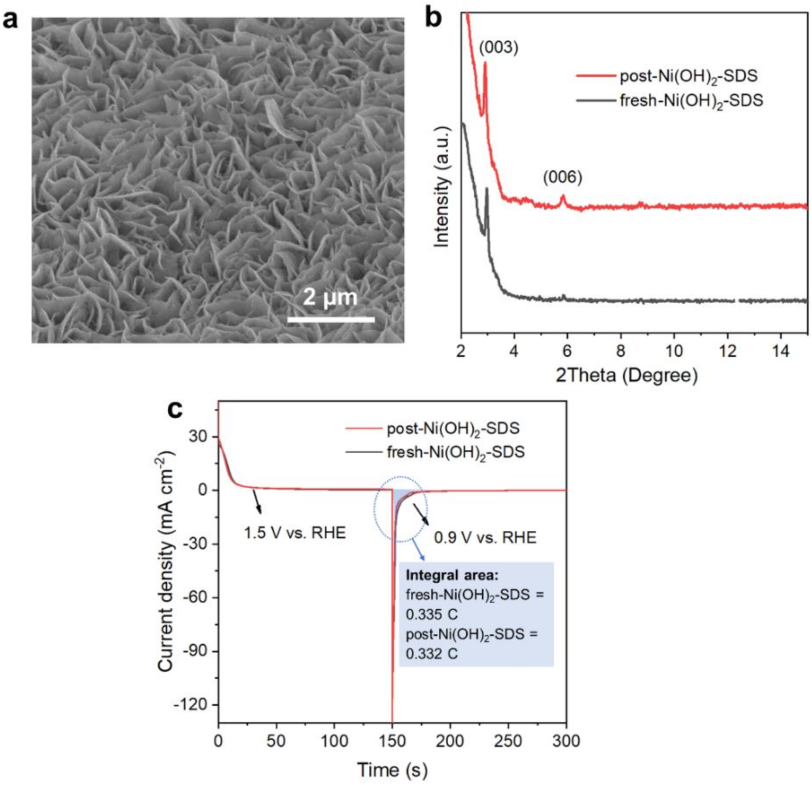

Supplementary Fig. 12 Characterizations of Ni(OH)2-SDS. **a**, SEM image of the post-Ni(OH)2-SDS. **b**, XRD pattern for the fresh and post-Ni(OH)2-SDS. The post Ni(OH)2-SDS catalyst was obtained after electrooxidation of cyclohexanone for 10 batches (overall 20 hours). The reaction conditions are 1.5 V vs RHE in 0.5 M KOH with 0.4 M cyclohexanone. **c**, Pulsed I-t curves of fresh and post-Ni(OH)2-SDS by oxidization of the samples at 1.5 V vs RHE in 0.5 M KOH for 150 s, followed by reduction at 0.9 V vs RHE for another 150 s.

The number of active Ni sites of Ni(OH)2-SDS after 20 hours of electrolysis (post-Ni(OH)2-SDS) were investigated by pulsed chronoamperometric (CA) measurement, which were measured by the calculation of the stored passing charge at 0.9 V vs RHE shown in the cathodic I-t curve (Supplementary Fig. 12c). The results show that the post-Ni(OH)2-SDS sample exhibits almost the same amount of passing charge as the fresh one (0.335 vs 0.332 C) under the same measured conditions, suggesting the active Ni sites in Ni(OH)2-SDS were maintained after 20 h of electrolysis, indicative of the stability of the catalyst.

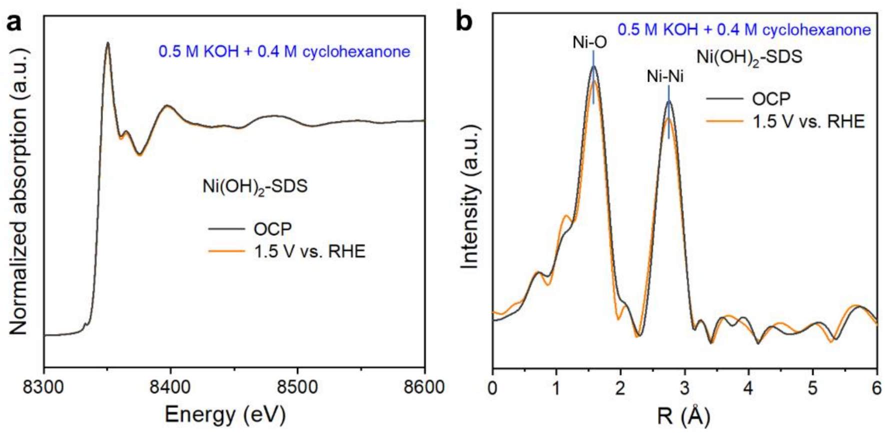

Supplementary Fig. 13 XANES spectra. **a**, Ni K-edge XANES spectra of $Ni(OH)_2$-SDS at open circuit potential (OCP) and after 900 s at 1.5 V vs RHE in a 0.5 M KOH with 0.4 M cyclohexanone at room temperature. **b**, The corresponding EXAFS Fourier transform (FT) spectra. After applying the potential (1.5 V vs RHE), the white line of the Ni K-edge XANES spectrum of $Ni(OH)_2$-SDS almost coincides with that under OCP. Moreover, R-space spectra show that the Ni−O and Ni-Ni(O bridged) bonds of $Ni(OH)_2$-SDS were only slightly weakened after applying the potential (1.5 V vs RHE), suggesting that the $Ni(OH)_2$ phase was largely maintained during the reaction, thus the interlayer SDS can be stabilized in $Ni(OH)_2$ interlayer.

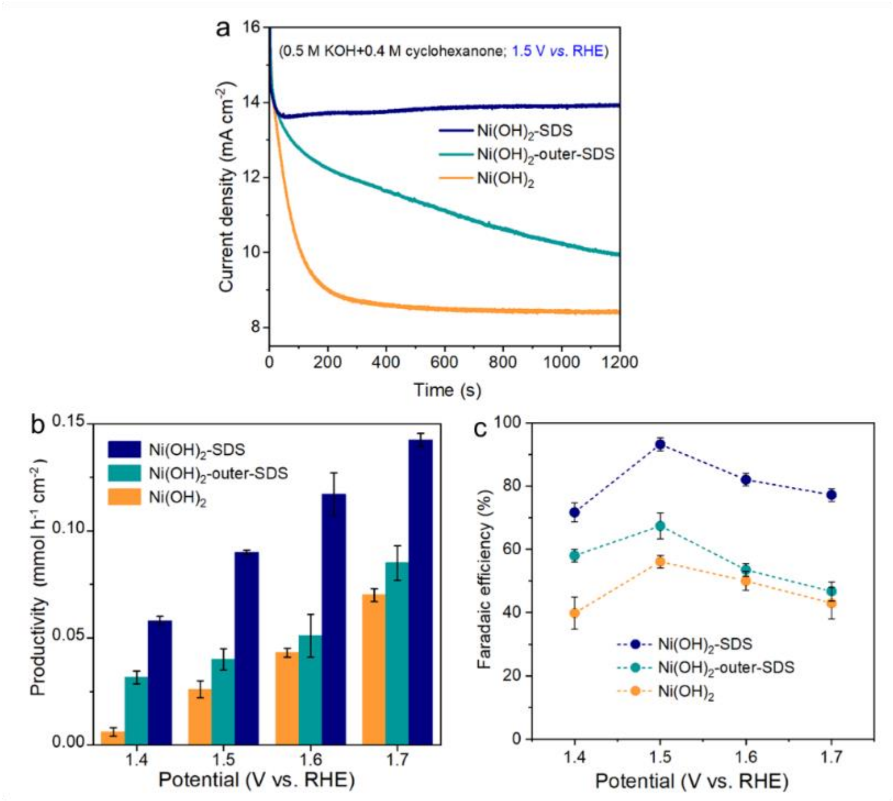

Supplementary Fig. 14 Electrochemical performances. a, *I-t* curves of cyclohexanone oxidation in 0.5 M KOH with 0.4 M cyclohexanone over $Ni(OH)_2$, $Ni(OH)_2$ with 5 mM SDS (denoted as $Ni(OH)_2$-outer-SDS) and $Ni(OH)_2$-SDS at 1.5 V vs RHE. **b**, Adipic acid productivity and **c**, FE of cyclohexanone oxidation in 0.5 M KOH with 0.4 M cyclohexanone over $Ni(OH)_2$, $Ni(OH)_2$-outer-SDS and $Ni(OH)_2$-SDS at different reaction potentials. The error bars represent the standard deviation of three parallel repeated results which is within 5%, the corresponding data represent the average values.

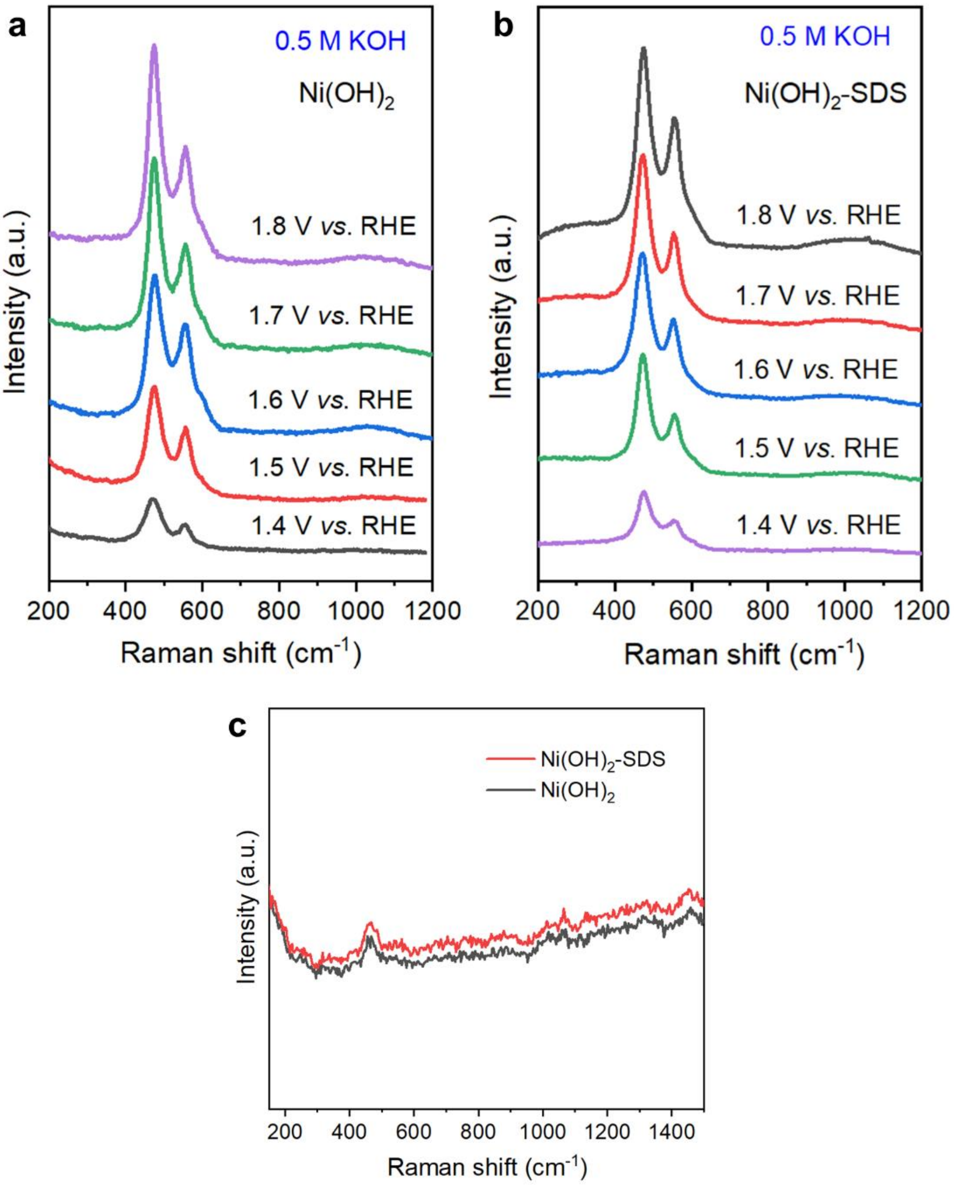

Supplementary Fig. 15 In situ Raman spectra. In situ Raman spectra of **a**, pure $Ni(OH)_2$ and **b**, $Ni(OH)_2$-SDS in 0.5 M KOH. **c**, Raman spectra of the pristine $Ni(OH)_2$ and $Ni(OH)_2$-SDS samples.

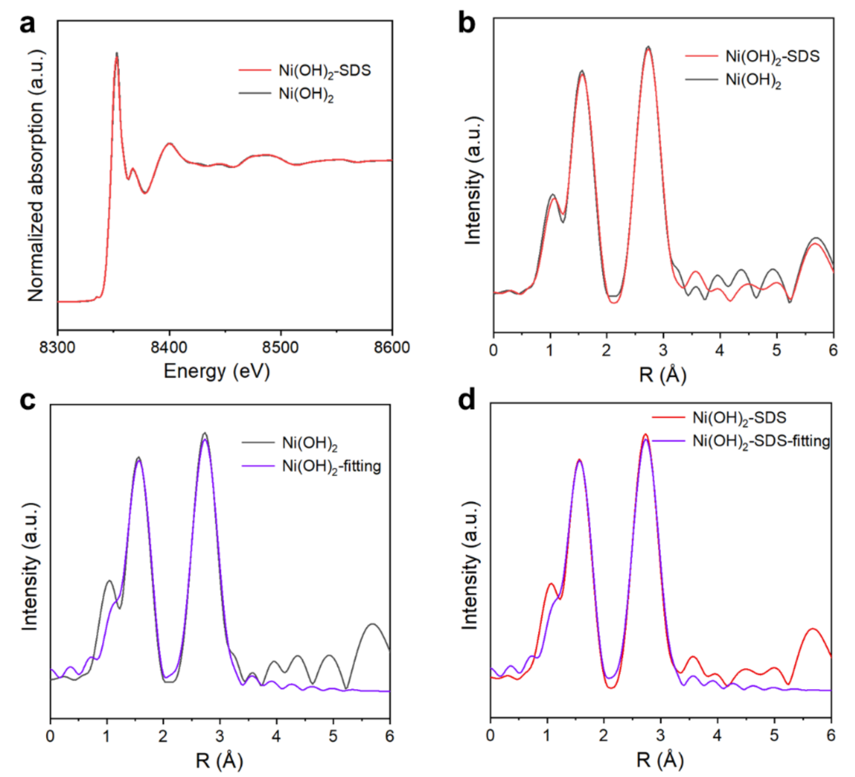

Supplementary Fig. 16 XANES spectra. a, Normalized XANES adsorption profiles and **b**, EXAFS Fourier transform (FT) spectra of the Ni K-edge XANES spectra of $Ni(OH)_2$-SDS and $Ni(OH)_2$. The corresponding fitting data of **c**, $Ni(OH)_2$ and **d**, $Ni(OH)_2$-SDS. The white line of the Ni K-edge XANES spectrum of $Ni(OH)_2$-SDS coincides with that of pure $Ni(OH)_2$. Moreover, R-space spectra show that the Ni−O and Ni-Ni(O bridged) bonds of $Ni(OH)_2$-SDS and $Ni(OH)_2$ were same, indicating that SDS intercalation would not significantly impact the initial structure of $Ni(OH)_2$.

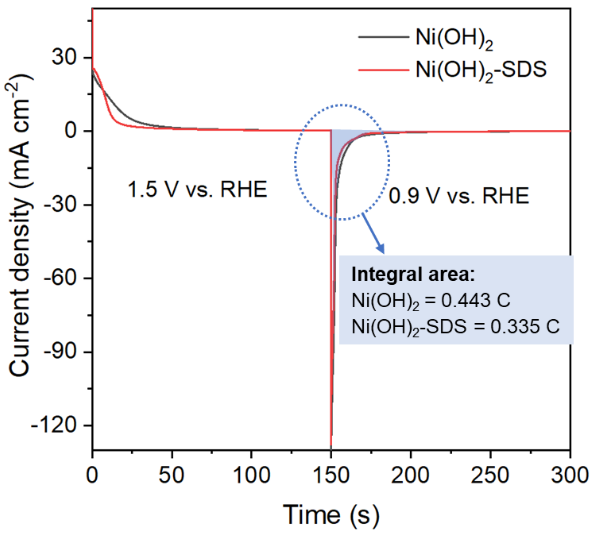

Supplementary Fig. 17 Pulsed CA measurements. Pulsed I-t curves of $Ni(OH)_2$-SDS and pure $Ni(OH)_2$ by oxidizing the catalyst at 1.5 V vs RHE in 0.5 M KOH for 150 s, and then reducing it at 0.9 V vs RHE for 150 s.

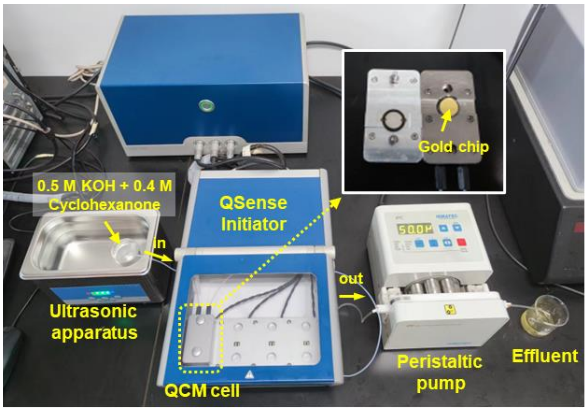

Supplementary Fig. 18 Photos of the QCM device. The QCM measurements were operated in a flow cell in a QSense Initiator. The QCM test was initiated for ~200 s in 0.5 M KOH to reach a steady status. Then 0.4 M cyclohexanone was added into the electrolyte, which was ultrasonicated to form emulsion and was then pumped into the QCM cell. Since the QCM test was performed in a continuous flow mode, requiring only a few seconds for transporting cyclohexanone solution to the QCM cell, thus the phase separation can be largely neglected during the process.

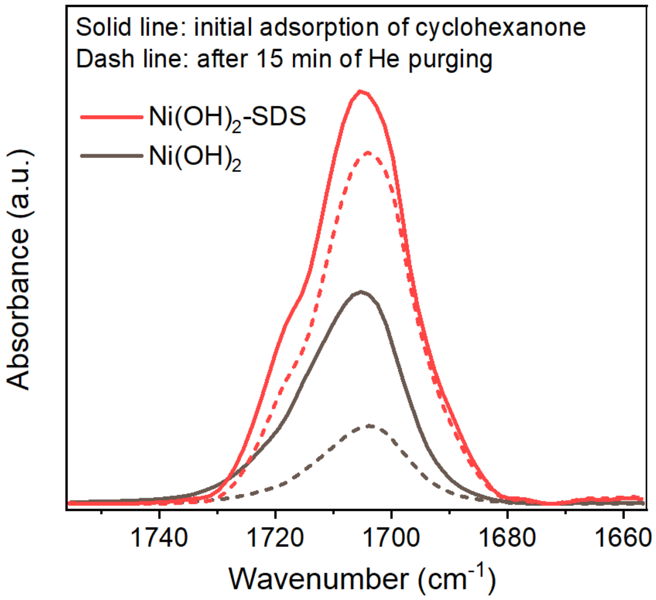

Supplementary Fig. 19 In situ FTIR spectra. FTIR spectra of $Ni(OH)_2$-SDS and pure $Ni(OH)_2$ after cyclohexanone adsorption. When cyclohexanone was introduced into the cell with $Ni(OH)_2$-SDS or $Ni(OH)_2$, the FTIR spectra show typical peak at 1713 cm^{-1}, which corresponds to the C=O stretching vibration of cyclohexanone.[1] The peak intensity of cyclohexanone on $Ni(OH)_2$-SDS is ~2-fold higher than that on $Ni(OH)_2$, indicating more cyclohexanone molecules are adsorbed over $Ni(OH)_2$-SDS. After purging with He for 15 min, the intensity of C=O peak on $Ni(OH)_2$ became weaker significantly, while it was highly maintained with only weak attenuation over $Ni(OH)_2$-SDS, indicating a stronger adsorption of cyclohexanone on $Ni(OH)_2$-SDS.

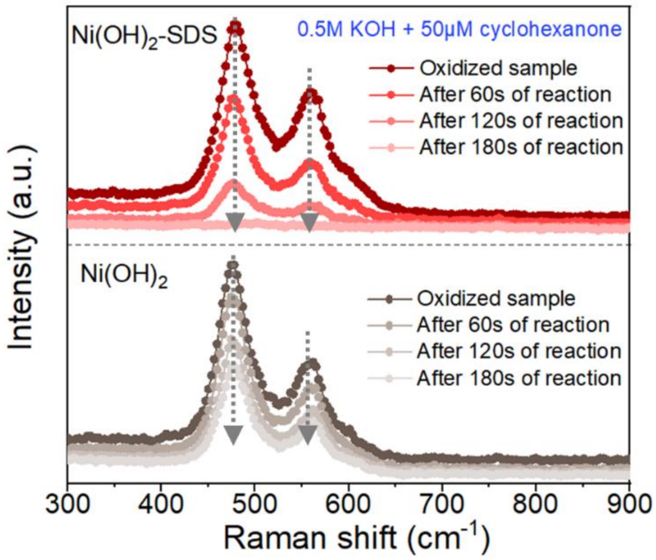

Supplementary Fig. 20 In situ Raman spectra. Raman spectra of the reduction process of the oxidized $Ni(OH)_2$-SDS and pure $Ni(OH)_2$ by cyclohexanone. The catalysts were firstly electrooxidized in 0.5 M KOH at 1.5 V for 60 s to generate NiOOH species (denoted as oxidized sample). Then, the oxidized samples were rapidly transferred into 0.5 M KOH with 50 µM cyclohexanone, allowing the reduction from NiOOH to $Ni(OH)_2$ by cyclohexanone. Raman spectra were collected at 60, 120 and 180 s, respectively.

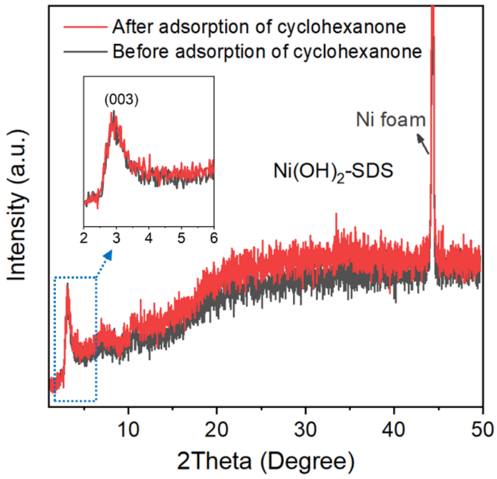

Supplementary Fig. 21 Characterizations of $Ni(OH)_2$-SDS. XRD patterns of $Ni(OH)_2$-SDS before and after adsorption of cyclohexanone. The $Ni(OH)_2$-SDS sample was immersed in a 0.5 M KOH solution with 0.4 M cyclohexanone, holding for 30 min to ensure cyclohexanone adsorption.

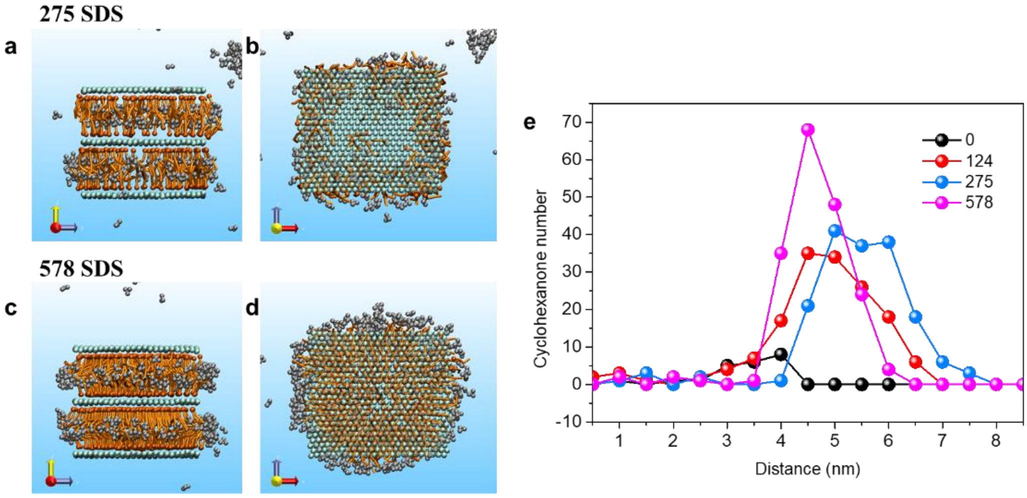

Supplementary Fig. 22 Coarse-grained molecular dynamic simulations of cyclohexanone diffusion behaviors. **a**, Side and **b**, top views of snapshot at 200 ns in $Ni(OH)_2$-SDS with 275 SDS molecules. **c**, Side and **d**, top views of snapshot at 200 ns in $Ni(OH)_2$-SDS with 578 SDS molecules. **e**, Cyclohexanone molecules distribution at different locations by comparing different numbers of SDS molecules in the $Ni(OH)_2$ (four directions from bulk solution to the center of catalysts are counted). Color codes: $Ni(OH)_2$ (green), SDS (orange), cyclohexanone (silver).

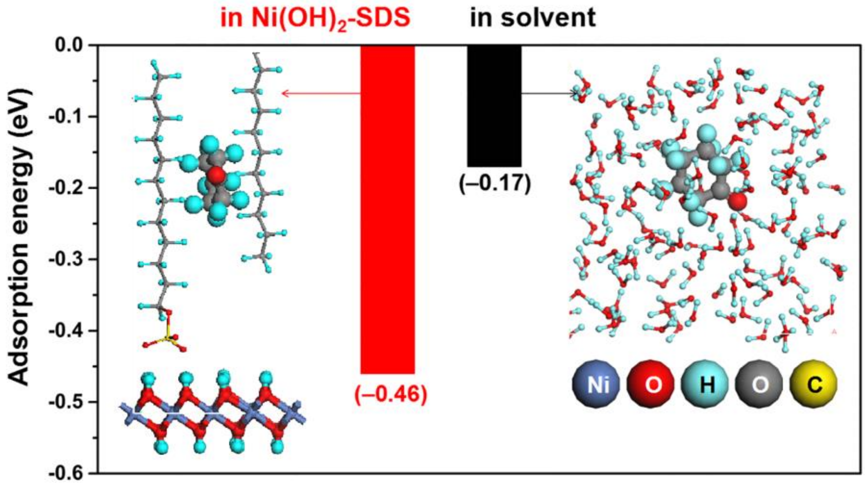

Supplementary Fig. 23 Adsorption energies. Adsorption energies of cyclohexanone in $Ni(OH)_2$-SDS and in solvent, calculated by spin-polarized DFT. The corresponding geometries are also displayed. The color codes are provided in the inset.

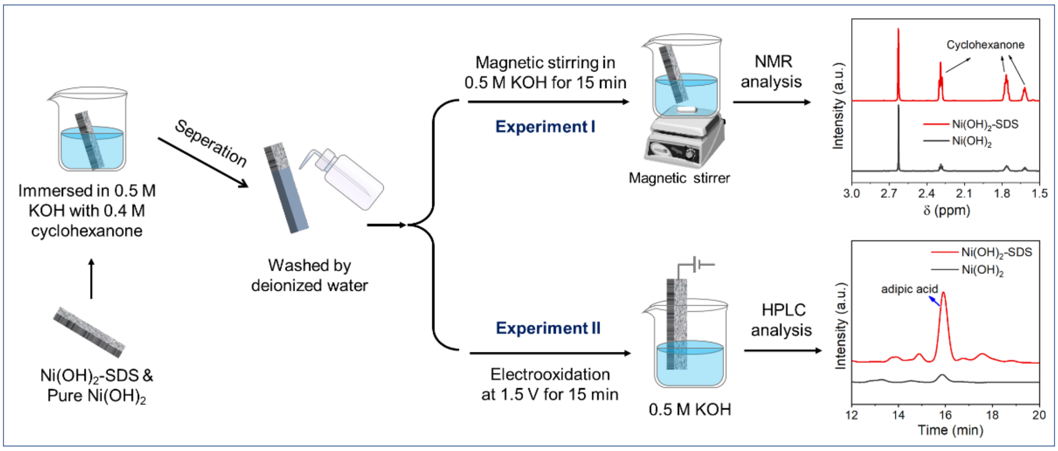

Supplementary Fig. 24 Cyclohexanone adsorption and release over catalysts. Schematic diagrams of the procedure for cyclohexanone adsorption over catalysts, the following release (Experiment I) and electrooxidation (Experiment II) processes, and the analysis results.

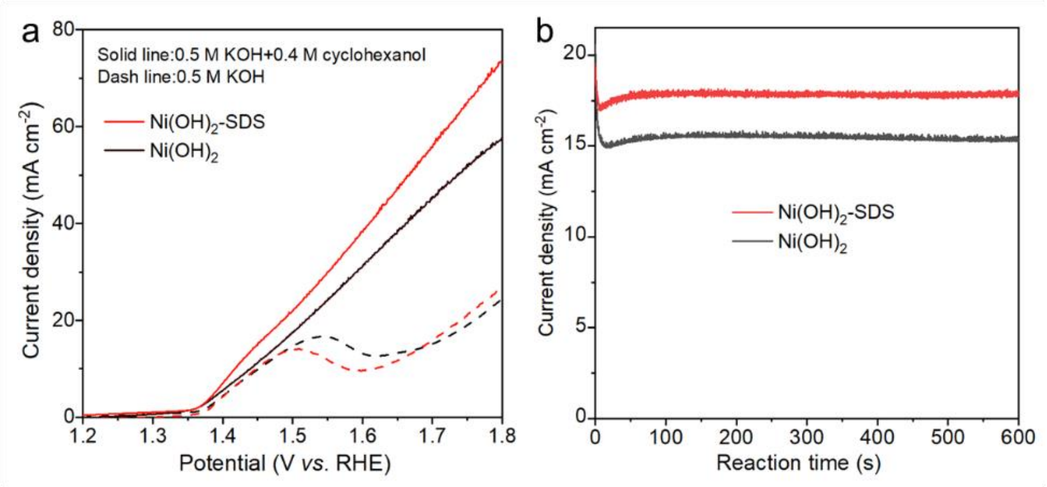

Supplementary Fig. 25 Electrochemical performances of cyclohexanol. a, LSV curves of $Ni(OH)_2$-SDS and pure $Ni(OH)_2$ catalysts at scan rate of 10 mV s^{-1}. **b**, *I-t* curves of cyclohexanol electrooxidation over $Ni(OH)_2$-SDS and $Ni(OH)_2$ at 1.5 V vs RHE in 0.5 M KOH with 0.4 M cyclohexanol.

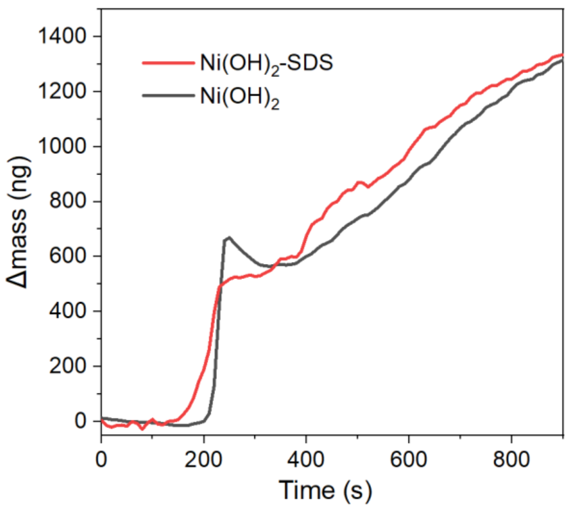

Supplementary Fig. 26 QCM results of cyclohexanol. QCM mass response over $Ni(OH)_2$-SDS and pure $Ni(OH)_2$ in 0.5 M KOH before and after adding 0.4 M cyclohexanol. The quartz crystal microbalance (QCM) results show that $Ni(OH)_2$-SDS and $Ni(OH)_2$ exhibit almost similar adsorption ability for cyclohexanol.

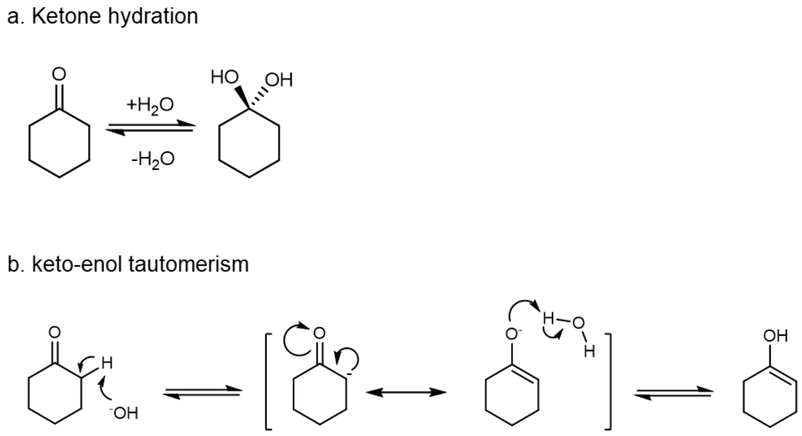

Supplementary Fig. 27 Reversible conversion of cyclohexanone in base. a, Reversible cyclohexanone hydration to geminal diol. **b**, Keto-enol automerization of cyclohexanone in alkaline electrolyte

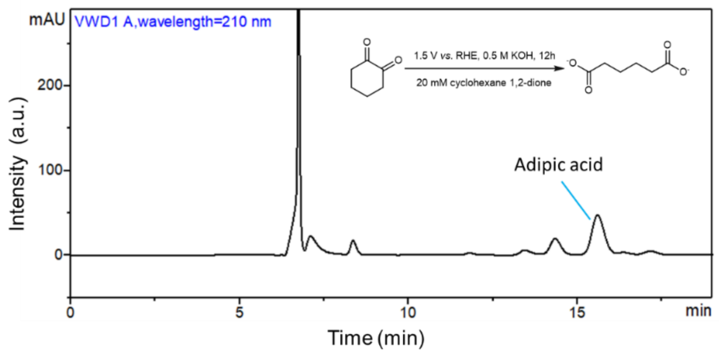

Supplementary Fig. 28 Cyclohexane 1,2-dione oxidation. HPLC spectra of the oxidation products for cyclohexane 1,2-dione over $Ni(OH)_2$-SDS. Reaction conditions: 1.5 V vs RHE, 0.5 M KOH with 20 mM cyclohexane 1,2-dione, 12 h.

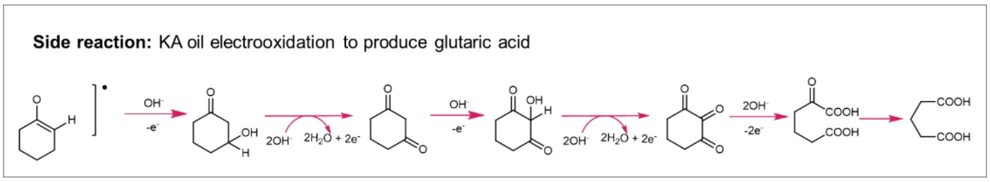

Supplementary Fig. 29 A plausible reaction mechanism for glutaric acid formation as the side reaction. Glutaric acid was observed over $Ni(OH)_2$-SDS and $Ni(OH)_2$ in the HPLC spectra at high potentials (Supplementary Fig. 8), which was probably generated through the formation of cyclohexane 1,3-dione intermediate during cyclohexanone oxidation.[2]

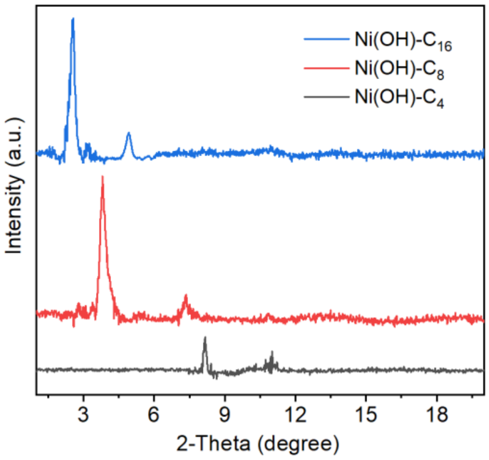

Supplementary Fig. 30 Characterizations of different catalysts. XRD patterns of $Ni(OH)_2$-C_4, $Ni(OH)_2$-C_8 and $Ni(OH)_2$-C_{16} samples.

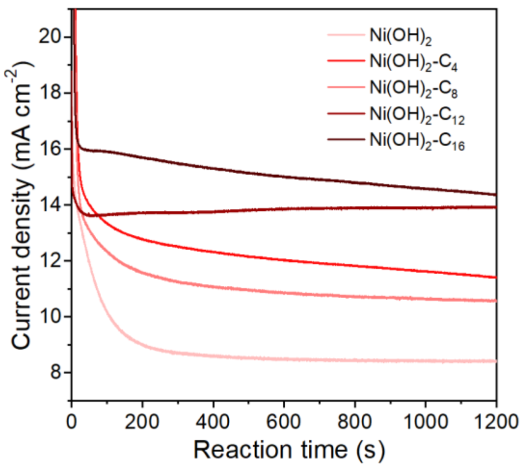

Supplementary Fig. 31 Electrochemical performances. *I-t* curves of Ni(OH)$_2$-C$_n$ samples in 0.5 M KOH with 0.4 M cyclohexanone at 1.5 V vs RHE.

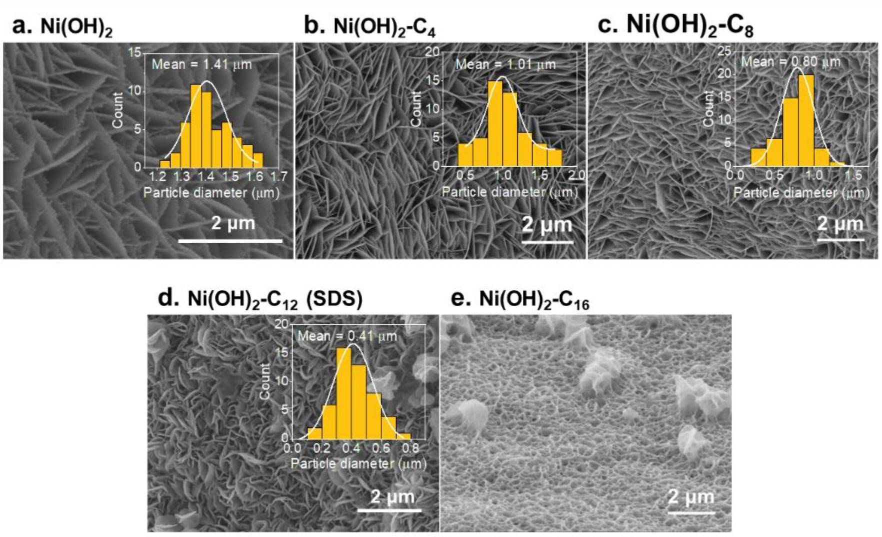

Supplementary Fig. 32 SEM images of Ni(OH)$_2$ and Ni(OH)$_2$-C$_n$ samples. SEM images of **a**, Ni(OH)$_2$, **b**, Ni(OH)$_2$-C$_4$, **c**, Ni(OH)$_2$-C$_8$, **d**, Ni(OH)$_2$-C$_{12}$, **e**, Ni(OH)$_2$-C$_{16}$. Insets in **a-d** are the size distribution of Ni(OH)$_2$ nanosheets with different carbon-chain lengths measured based on the SEM images. The results show that the average diameter of Ni(OH)$_2$ nanosheets was decreased from 1.4 μm (for pure Ni(OH)$_2$) to ~0.4 μm (for Ni(OH)$_2$-SDS), and the nanosheet structure of Ni(OH)$_2$-C$_{16}$ was collapsed which make it difficult to measure the diameter.

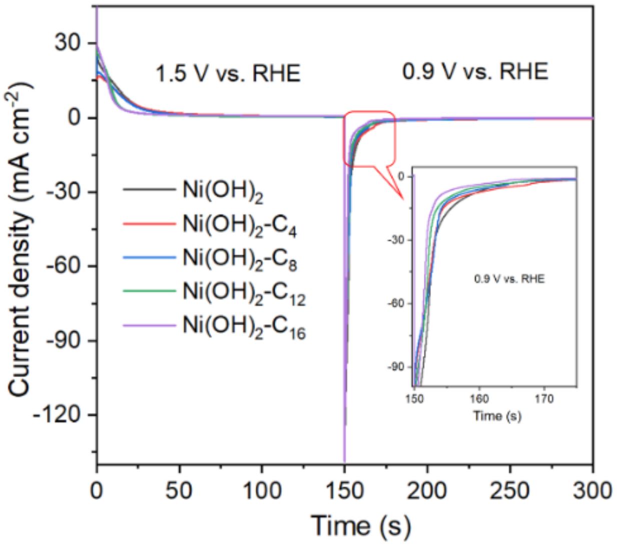

Supplementary Fig. 33 Pulsed CA measurements. Pulsed I-t curves of pure $Ni(OH)_2$ and $Ni(OH)_2$-C_n samples. The catalyst was first oxidized at 1.5 V vs RHE in 0.5 M KOH, and then reduced at 0.9 V vs RHE. Then, the reducible Ni sites can be measured by calculating the passing charge stored in cathodic I-t curve at 0.9 V vs RHE. The passing charge can be obtained by integration of the current response to the cathodic voltage pulses, by using the method proposed in the previous report.[3]

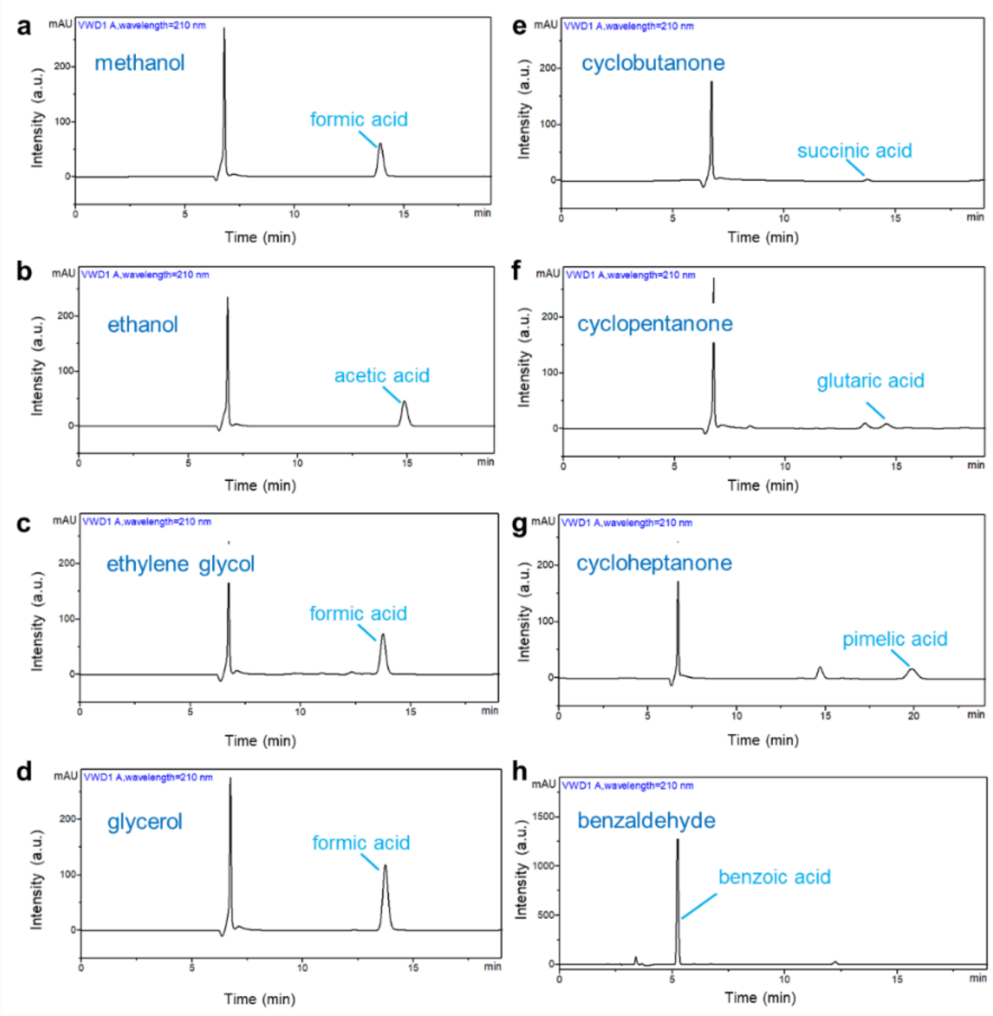

Supplementary Fig. 34 HPLC spectra of the oxidation products for different substrates. a, methanol, **b**, ethanol, **c**, ethylene glycol, **d**, glycerol, **e**, cyclobutanone, **f**, cyclopentanone, **g**, cycloheptanone, **h**, benzaldehyde. Reaction conditions: 1.5 V vs RHE, 0.5 M KOH with 0.4 M different substrate, 1 h.

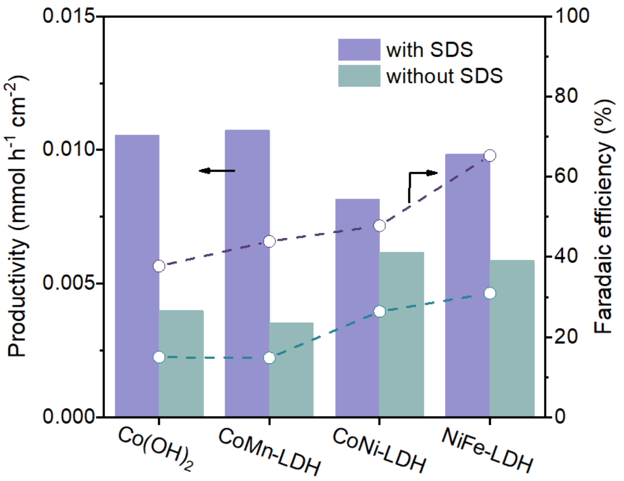

Supplementary Fig. 35 Electrochemical performances of different layered materials. Adipic acid productivity and FE over different catalysts modified with or without SDS in 0.5 M KOH with 0.4 M cyclohexanone at 1.5 V vs RHE.

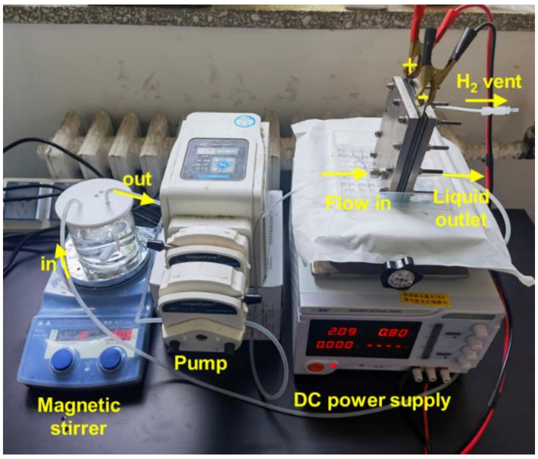

Supplementary Fig. 36 Two-electrode flow electrolyzer system. Photo of the device for electrochemical tests in two-electrode membrane-free flow electrolyzer. The concentration of cyclohexanone was 0.05 M in KOH electrolyte, lower than its solubility (~0.29 M), thus the cyclohexanone can be dissolved in the electrolyte without encountering the problem associated with phase separation.

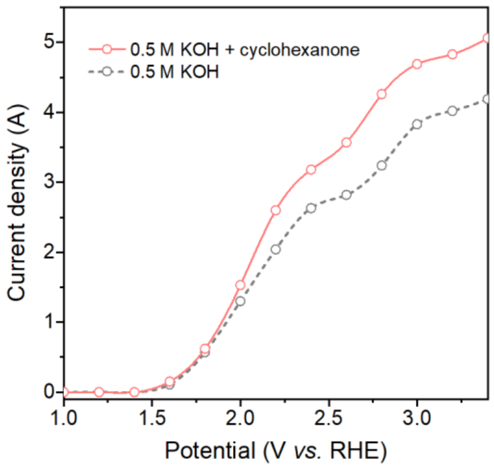

Supplementary Fig. 37 Electrochemical performances in the electrolyzer. LSV curves of Ni(OH)$_2$-SDS in the membrane-free flow electrolyzer in 0.5 M KOH or 5 M KOH with 50 mM cyclohexanone.

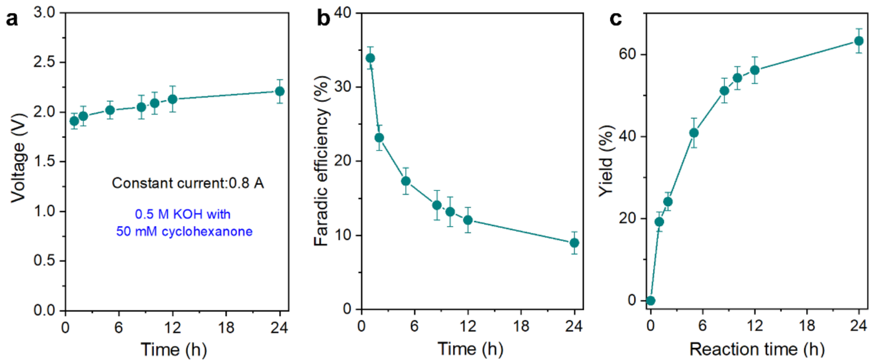

Supplementary Fig. 38 Electrochemical performances in the electrolyzer. a, Voltage-time curves of Ni(OH)$_2$-SDS in the membrane-free flow electrolyzer at 0.8 A in 0.5 M KOH with 50 mM. Corresponding **b**, FE and **c**, Yield of adipic acid. To demonstrate the decreased catalytic performance (FE and formation rate of adipic acid) is not due to the instability of the Ni(OH)$_2$-SDS, we carried out the reaction in batch reactor for 24 hours, and then reused the catalyst three times by replenishing fresh electrolyte. The error bars represent the standard deviation of three independent measurements, which is within 10%. The data represent the average value. The results show that the performance of Ni(OH)$_2$-SDS can be restored, suggesting that Ni(OH)$_2$-SDS is stable under our reaction conditions

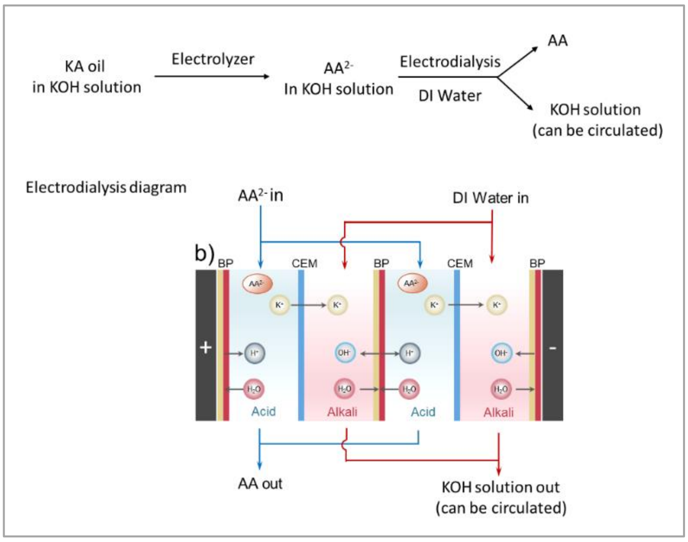

Supplementary Fig. 39 Product separation. The proposed electrocatalysis-coupling-electrodialysis (EC-ED) system. Adipic acid (AA) is in the form of carboxylate (denoted as AA^{2-}) in the 0.5 M KOH considering its pKa values (pKa_1 = 4.42, pKa_2 = 5.41) are lower than the pH value (13.7) of the electrolyte under the catalytic conditions.

Supplementary Tables

Supplementary Table 1. Investigations of the molar ratio of SDS to Ni in $Ni(OH)_2$-SDS before and after 20-hours reaction at 1.5 V vs RHE.[a]

$Ni(OH)_2$-SDS	Molar ratio of SDS : Ni (%)	Weight ratio of SDS : $Ni(OH)_2$ (wt%)
Before reaction	1 : 14.0	1 : 4.50
After 20-hours reaction	1 : 14.3	1 : 4.56

[a] The molar ratio of SDS:Ni in the post $Ni(OH)_2$-SDS was only slightly decreased, implying that most of the SDS molecules were stabilized in the $Ni(OH)_2$ interlayer after the catalytic reactions.

Supplementary Table 2. Solubilities of cyclohexanol, cyclohexanone and adipic acid in water and base.

Substrate	Solubility (g L^{-1})		
	In water (20 ^oC) from MSDS	In water (20 ^oC) by measurements	In 0.5 M KOH (20 ^oC) by measurements
**Cyclohexanone (K)**	70	70.2	25.7
**Cyclohexanol (A)**	36	34.1	29.4
**Adipic acid (AA)**	23	27.2	miscible

Supplementary Table 3. The data of thermochemical data (standard reaction Gibbs energy of formation).[a,b]

Compounds	ΔG^0_f (kJ mol^{-1})
H_2O	−237.1
Cyclohexanone	−90.79
Adipic acid	−135.13

[a] The thermochemical data are from the Handbook of Chemistry and Physics.

[b] $\Delta G^0_{reaction} = \Delta G^0_f(\text{Adipic acid}) - \Delta G^0_f(\text{Cyclohexanone}) - 3\Delta G^0_f(H_2O) = 666.96\ kJ.mol^{-1}$

Supplementary Table 4. Reproducibility of $Ni(OH)_2$-SDS catalysts from three batches.[a]

$Ni(OH)_2$-SDS	Adipic acid productivity ($\mu mol\ cm^{-2}\ h^{-1}$)	Faradic efficiency (%)
Batch 1	89.5	91.6
Batch 2	90.4	93.2
Batch 3	92.8	94.8

[a] The catalytic reaction was performed in 0.5 M KOH with 0.4 M cyclohexanone at 1.5 V vs RHE in 1 hour. The original catalytic results of product distribution are shown in Supplementary Fig. 10. Note: $Ni(OH)_2$-SDS samples from different batches exhibit similar catalytic performance, achieving adipic acid productivity of 90 ± 3 $\mu mol\ cm^{-2}\ h^{-1}$ and FE of 93 ± 2 %, demonstrating the good reproducibility of the $Ni(OH)_2$-SDS catalyst.

Supplementary Table 5. Summary of electrocatalytic KA oil oxidation to produce adipic acid over different catalysts.

**Catalyst**	**Electrolyte**	**Substrate**	**Reaction conditions**	**Conv. (%)**	**Yield of adipic acid (%)**	**Ref.**
CoMnOOH/Ni foam	1 M KOH	20 mM cyclohexanol	1.45 V vs RHE, RT, 17 h	~100	64.2	4
NiOOH	1 M NaOH	150 mM cyclohexanol	6 mA cm^{-2} 10 °C	~100	46.7	5
		150 mM cyclohexanol		70.2	52.3	
$Ni(OH)_2$-SDS/Ni foam	0.5 M KOH	20 mM cyclohexanol	1.5 V vs RHE, RT, 25 h	~100	86.5	This work
		20 mM cyclohexanone	1.5 V vs RHE, RT, 16 h	~100	84.0	

Supplementary Table 6. Concentrations of SDS in the electrolyte before and after 20-hours reaction at 1.5 V vs RHE.[a,b,d]

	**Before reaction**	**After 20-hours reaction**
Concentration of SDS in electrolyte (mg/L)	0.0	0.2
Leaching ratio (%)[c]	0.0	0.8

[a] Reaction conditions: $Ni(OH)_2$-SDS (1 cm^2) in 25 mL of 0.5 M KOH with 0.4 M cyclohexanone;
[b] ICP-OES conditions: Agilent ICP-OES 725 ES. RF Power: 1.20 kW, Plsama flow: 15.0 L/min, Nebulizer flow: 0.75 L/min, Sample uptake delay: 10 s, Replicate read time: 15 s, Replicates: 3.
[c] The total SDS concentration in the $Ni(OH)_2$-SDS sample was measured to be 26.2 mg/L.
[d] The concentration of the S species in the electrolyte were measured before and after the catalytic reactions. The results show that the leaching ratio of SDS was insignificant (~0.8 %) after 20 hours of reaction, indicating that only a small amount of SDS detached from the Ni sites after long-term reaction.

Supplementary Table 7. EXAFS fitting parameters at the Ni *K*-edge for different Ni-based catalysts and references.

**Sample**	Shell	N [a]	R (Å) [b]	σ^2 ($Å^2 \cdot 10^{-3}$) [c]	ΔE^0 (eV) [d]	R factor (%)
$Ni(OH)_2$	Ni–O	6.3(±0.9)	2.03(±0.02)	7.2	-6.5	0.9
	Ni–Ni (O bridged)	6.1(±1.1)	3.10(±0.02)	7.9	0.6	
$Ni(OH)_2$-SDS	Ni–O	5.9(±0.7)	2.03(±0.02)	7.6	-5.9	0.6
	Ni–Ni (O bridged)	6.2(±0.8)	3.10(±0.02)	8.4	-1.3	
NiO	Ni–O	6.6(±1.1)	2.09(±0.02)	9.2	-7.3	1.7
	Ni–Ni (O bridged)	11.4(±1.5)	2.96(±0.01)	11.3	-7.5	
Ni foil (25°C)	Ni–Ni (metal state)	12	2.48	5.9	6.8	1.0

[a] N: coordination number; [b] R: bond distance; [c] σ^2: Debye-Waller factor; [d] ΔE_0: the inner potential correction.
R factor: goodness of fit. S_0^2, 0.90, was obtained from the experimental EXAFS fitting over NiO reference with known crystallographic value, which was then used to all the samples.
Note: The EXAFS results show that the $Ni(OH)_2$ and $Ni(OH)_2$-SDS have similar coordination structure of Ni in terms of coordination number and bond distance, indicating that SDS intercalation would not significantly impact the initial structure of $Ni(OH)_2$.

Supplementary Table 8. Comparison of the mass loading, ECSA, productivity and ECSA-normalized productivity of adipic acid over $Ni(OH)_2$-SDS and $Ni(OH)_2$ catalysts.

Sample	Entry	Mass loading (mg/cm^2)	ECSA[a] ($mF\ cm^{-2}$)	Productivity[b] ($\mu mol\ cm^{-2}\ h^{-1}$)	ECSA-normalized productivity[c] ($\mu mol\ mF^{-1}\ h^{-1}$)
$Ni(OH)_2$	1	2.69	0.79	22.9	29.0
	2	2.80	0.84	25.1	29.9
	3	2.91	0.95	29.3	30.8
$Ni(OH)_2$-SDS	1	3.62	1.35	89.5	66.3
	2	3.88	1.37	90.4	65.9
	3	4.20	1.49	92.8	62.3

[a] ECSA values of $Ni(OH)_2$-SDS and $Ni(OH)_2$ samples were determined by measuring the capacitive current associated with double-layer charging from the scan-rate dependence of CVs. The ECSA value was measured on the same working electrode and electrolyte (0.5 M KOH). The potential window of CVs was (-0.45)–(0.45) V vs Ag/AgCl, and the scan rates were 50, 75, 100, 125, 150, 175, 200 mV s^{-1}. The double layer capacitance (C_{dl}) was estimated by plotting the charging current density difference ($\Delta j = j_a - j_c$) at 0 V vs Ag/AgCl against the scan rate. The slope is twice of C_{dl}.

[b] The catalytic reaction was performed in 0.5 M KOH with 0.4 M cyclohexanone at 1.5 V vs RHE in 1 hour.

[c] The ECSA value is positively correlated with the mass loading. We think the catalytic activity normalized by ECSA would be reasonable to compare the electrocatalytic performance. To demonstrate the possibility, $Ni(OH)_2$ samples prepared independently (albeit with different mass loading) were subject to electrooxidation of cyclohexanone. The obtained adipic acid productivity was then normalized by ECSA, showing comparable values. We then evaluated $Ni(OH)_2$-SDS activity by using the same normalization method, affording the same conclusion. These results suggest that the ECSA-normalized productivity can be tentatively used to compare the electrocatalytic performance over different catalysts, and the effect of different mass loading can be safely decoupled. As a result, the ECSA-normalized productivity of $Ni(OH)_2$-SDS is ~2.2-fold higher than that of $Ni(OH)_2$, indicating the promoting effect of SDS for electrooxidation cyclohexanone.

Supplementary Table 9. Comparison of the mass loading, numbers of the reducible Ni sites, productivity and reducible Ni sites-normalized productivity of adipic acid over $Ni(OH)_2$-SDS and $Ni(OH)_2$ catalysts.

Sample	Entry	Mass loading (mg/cm^2)	Numbers of the reducible Ni sites[a] (C)	Productivity of adipic acid[b] ($\mu mol\ cm^{-2}\ h^{-1}$)	Reducible Ni sites-normalized productivity[c] ($\mu mol\ cm^{-2}\ h^{-1}\ C^{-1}$)
$Ni(OH)_2$	1	2.69	0.435	22.9	52.6
	2	2.80	0.443	25.1	56.7
	3	2.91	0.462	29.3	63.4
$Ni(OH)_2$-SDS	1	3.62	0.331	89.5	270.5
	2	3.88	0.335	90.4	269.9
	3	4.20	0.340	92.8	272.9

[a] The numbers of the reducible Ni sites were determined by pulsed chronoamperometric (CA) measurement shown in the Supplementary Fig. 17.

[b] The catalytic reaction was performed in 0.5 M KOH with 0.4 M cyclohexanone at 1.5 V vs RHE in 1 hour.

[c] The increased amplitude of productivity by reducible sites normalization (4.8-fold) is larger than the one by ECSA normalization (2.2-fold), with the former possibly representing a more accurate metric for catalytic comparison due to only the reducible sites of Ni are accounted.

Supplementary Table 10. The interlayer space of different $Ni(OH)_2$-C_n samples that detected by XRD and the length of interlayer ligands.

Sample	Interlayer space d (nm)[a]	Length of the ligand (nm)
**$Ni(OH)_2$**	0.76	/
**$Ni(OH)_2$-C_4**	1.11	0.9
**$Ni(OH)_2$-C_8**	2.32	1.5
**$Ni(OH)_2$-C_{12}**	2.95	1.8
**$Ni(OH)_2$-C_{16}**	3.30	2.5

[a] The interlayer space was calculated based on the (003) reflection of XRD following the equation: $2d\sin\theta = n\lambda$ (n=1, λ=0.15406). The theoretical interlayer distance can be calculated as: $d_{calcualted} = d_{layer} + d_{inter}$, where d_{layer} represents the thickness of the $Ni(OH)_2$ sheet (~0.49 nm) and d_{inter} includes the length of intercalated species and absorptive water in the interlayer.

Supplementary Table 11. Comparison of the mass loading, ECSA, productivity and ECSA-normalized productivity over $Ni(OH)_2$ and $Ni(OH)_2$-C_n samples.

Sample	Mass loading (mg/cm^2)	ECSA[a] ($mF\ cm^{-2}$)	Productivity of adipic acid[b] ($\mu mol\ cm^{-2}\ h^{-1}$)	ECSA-normalized productivity[c] ($\mu mol\ mF^{-1}\ h^{-1}$)
$Ni(OH)_2$	2.80	0.84	25.1	29.9
$Ni(OH)_2$-C_4	2.93	0.89	43.3	48.7
$Ni(OH)_2$-C_8	3.46	1.05	51.9	49.4
$Ni(OH)_2$-C_{12}(SDS)	3.88	1.37	90.4	65.9
$Ni(OH)_2$-C_{16}	3.51	1.15	83.2	72.3

[a] ECSA values of $Ni(OH)_2$ and $Ni(OH)_2$-C_n samples were determined by measuring the capacitive current associated with double-layer charging from the scan-rate dependence of CVs. The ECSA value was measured on the same working electrode and electrolyte (0.5 M KOH). The potential window of CVs was (-0.45)–(0.45) V vs Ag/AgCl, and the scan rates were 50, 75, 100, 125, 150, 175, 200 mV s^{-1}. The double layer capacitance (C_{dl}) was estimated by plotting the charging current density difference ($\Delta j = j_a – j_c$) at 0 V vs Ag/AgCl against the scan rate. The slope is twice of C_{dl}.

[b] The catalytic reaction was performed in 0.5 M KOH with 0.4 M cyclohexanone at 1.5 V vs RHE in 1 hour.

[c] After normalized by ECSA, the productivity of $Ni(OH)_2$-C_n samples are higher than that of pure $Ni(OH)_2$, indicating the promoting effect of interlayer SDS for electrooxidation cyclohexanone.

Supplementary Table 12. Comparison of the numbers of reducible Ni sites, productivity, reducible Ni sites-normalized productivity and FE of adipic acid over pure $Ni(OH)_2$ and $Ni(OH)_2$-C_n samples.

**Sample**	**Numbers of the reducible Ni sites[a] (C)**	**Productivity of adipic acid[b] (μmol cm^{-2} h^{-1})**	**Reducible Ni sites-normalized productivity[C] (μmol cm^{-2} h^{-1} C^{-1})**	**FE (%)**
$Ni(OH)_2$	0.443	25.1	56.7	54
$Ni(OH)_2$-C_4	0.429	43.3	100.9	71
$Ni(OH)_2$-C_8	0.382	51.9	135.9	74
$Ni(OH)_2$-C_{12}(SDS)	0.335	90.4	269.9	93
$Ni(OH)_2$-C_{16}	0.312	83.2	266.7	88

[a] The numbers of the reducible Ni sites were determined by pulsed chronoamperometric (CA) measurement shown in the Supplementary Fig. 31.

[b] The catalytic reaction was performed in 0.5 M KOH with 0.4 M cyclohexanone at 1.5 V vs RHE in 1 hour.

[c] The pure $Ni(OH)_2$ has more reducible Ni sites than $Ni(OH)_2$-C_n samples, revealing that the intercalation of SDS may hinder the exposure of Ni active sites over $Ni(OH)_2$ surface. The number of reducible Ni sites on $Ni(OH)_2$-C_{16} is relatively lower, probably due to the collapse of the nanosheet as observed by SEM. After normalizing the productivities of adipic acid by the numbers of reducible Ni sites, we can see the promoted productivity of $Ni(OH)_2$-C_n samples compared with pure $Ni(OH)_2$. In addition, the productivity is higher over $Ni(OH)_2$ with longer ligand, which is probably due to the larger adsorption capacity in the interlayer. Particularly, $Ni(OH)_2$-SDS sample exhibits the highest adipic acid productivity (269 μmol cm^{-2} h^{-1} C^{-1}, 4.8-fold higher than that of pure $Ni(OH)_2$), demonstrating the promoting effect of interlayer ligand for electrooxidation of cyclohexanone

Supplementary Notes

Supplementary Note 1

To determine the molar ratio of SDS to Ni on one side of $Ni(OH)_2$ layer, which corresponds to the percentage of SDS-coordinated Ni ion sites, a schematic structure of SDS-intercalated $Ni(OH)_2$ is needed based on the experimental results. The HRTEM image (Supplementary Fig. 5a) of a representative $Ni(OH)_2$-SDS shows that the nanosheet thickness is about 15 nm, implying that the $Ni(OH)_2$-SDS is composing of 6-layers of $Ni(OH)_2$, and SDS anions are intercalated on both sides of an individual $Ni(OH)_2$ layer in parallel, thus forming 10-layers of SDS (Supplementary Fig. 5b). Of note, this schematic structure is an estimated result based on limited numbers of nanosheets, and the layer numbers might be varied between different nanosheets. Based on the estimation, the molar ratio of SDS to Ni on one side of individual $Ni(OH)_2$ layer can be calculated based on the equation of 1/10 : 14/6, yielding 1:23.3. Namely, the percentage of SDS-coordinated Ni ion sites in the $Ni(OH)_2$-SDS catalysts is 1:23.3. This value is lower than what we expected, which might be attributed to the steric hinderance of SDS in the interlayer and the insufficient intercalation of the synthetic method.

Supplementary Note 2

The overall reactions (cyclohexanone oxidation coupled with HER, and overall water splitting) and the anodic and cathodic half-reactions are shown below, associated with the calculated standard reaction Gibbs energy (ΔG^0) and the cell voltage. ΔG^0 was calculated by the use of thermochemical data (Supplementary Table 3). Based on the equation of $\Delta G^0 = -zFE^0$ (where z is the number of electrons transferred (z = 6), E^0 represents the standard electromotive force), the cell voltage for the electrolysis of cyclohexanone oxidation coupled with HER is calculated to be 1.152 V. As a result, the standard electrode potential (φ^0) of anodic cyclohexanone oxidation is 1.152 V vs RHE, which is lower than that of water oxidation (1.23 vs RHE), suggesting that cyclohexanone oxidation is thermodynamically more favorable.

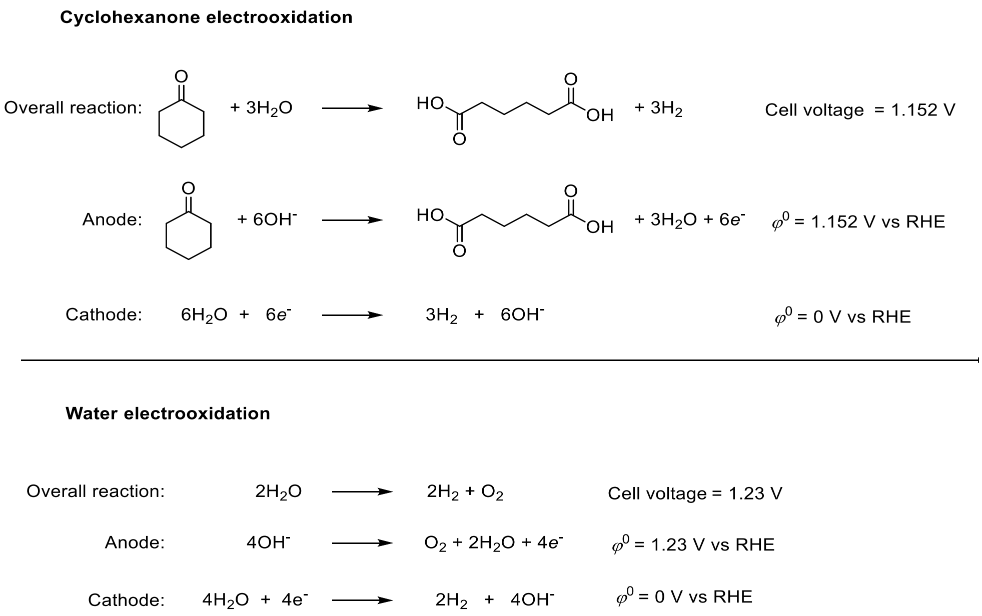

Supplementary Note 3

To study the stability of SDS in $Ni(OH)_2$, the post $Ni(OH)_2$-SDS after 20-hours reaction was characterized by series techniques including SEM, XRD, and ICP-OES. The reaction conditions were 0.5 M KOH with 20 mM cyclohexanone at 1.5 V vs RHE. As shown in the Supplementary Fig. 12a, the pristine nanosheet array structure of $Ni(OH)_2$-SDS was preserved. The distance between two layers in $Ni(OH)_2$ were maintained, which was estimated to be ~2.9 nm (Supplementary Fig. 12b), suggesting the SDS molecules were still intercalated in the $Ni(OH)_2$ after the catalytic reaction. Moreover, the molar ratio of SDS:Ni in the post $Ni(OH)_2$-SDS was only slightly decreased (from 1:14.0 to 1:14.3; Supplementary Table 1), implying that most of the SDS molecules were stabilized in the $Ni(OH)_2$ interlayer after the catalytic reactions. In addition, the concentration of the S species in the electrolyte were measured before and after the catalytic reactions (the ICP-OES results; Supplementary Table 6). The results show that the leaching ratio of SDS was insignificant (~0.8 %) after 20 hours of reaction, indicating that only a small amount of SDS detached from the Ni sites after long-term reaction. We ascribe the detached SDS, as the reviewer mentioned, to the SDS originally stabilized on Ni ion sites which were reconstructed to NiOOH, presenting only a small proportion of the total Ni sites considering their presence on the

edge sites.

Taken together, we speculate that only $Ni(OH)_2$ on outer surface or at edge of the nanosheets were converted to NiOOH, leaving the majority of $Ni(OH)_2$ structure being maintained, thus the SDS molecules can be stabilized in the interlayer.

Supplementary Note 4

Note that SDS molecules might be stabilized on both the outer surface and interlayer of $Ni(OH)_2$-SDS. To identify their independent contribution to the promoted electrooxidation of cyclohexanone, we prepared $Ni(OH)_2$ sample which is only modified with SDS on the outer surface ($Ni(OH)_2$-outer-SDS). The sample was made by adding SDS into the electrolyte in the presence of $Ni(OH)_2$ as the anode, then the negatively charged SDS would be spontaneously adsorbed on the outer surface of $Ni(OH)_2$ (positively charged) under electric field. Compared with pure $Ni(OH)_2$, the $Ni(OH)_2$-outer-SDS shows increased adipic acid productivity from 25 to 40 μmol cm^{-2} h^{-1} and higher FE from 56% to 67% at 1.5 V vs RHE (Supplementary Fig. 14). Nevertheless, these values are significantly lower than that of $Ni(OH)_2$-SDS (adipic acid productivity of 90 μmol cm^{-2} h^{-1} and FE of 93%), in which both outer surface and interlayer are modified with SDS. Moreover, the current density of $Ni(OH)_2$-outer-SDS was not stable as that of $Ni(OH)_2$-SDS for longer time reaction (Supplementary Fig. 14a), suggesting that SDS is more likely to be stabilized in the interlayer of $Ni(OH)_2$-SDS. Based on these results, we tentatively rationalize that the interlayer of $Ni(OH)_2$-SDS plays a more prominent role for the enhanced activity of cyclohexanone oxidation.

Supplementary Note 5

For the reason of the faster reduction rate of NiOOH by cyclohexanone over $Ni(OH)_2$-SDS compared with $Ni(OH)_2$, we propose that the electrooxidation of cyclohexanone over $Ni(OH)_2$ follows a two-step "electrochemical-chemical" mechanism.[6] Specifically, it involves electrochemical dehydrogenation of $Ni(OH)_2$ to NiOOH ($Ni^{2+}(OH)_2 + OH^- \rightarrow Ni^{3+}OOH + H_2O + e^-$), followed by the spontaneous

nucleophile dehydrogenation with organic compounds, cyclohexanone and its reaction intermediates in our study ($Ni^{3+}OOH + H_{Nu} + e^{-}_{Nu} \rightarrow Ni^{2+}(OH)_2$). Therefore, the reduction rate of the NiOOH-related peaks can reflect the activity of cyclohexanone oxidation. Owing to the enrichment by SDS, the supply of cyclohexanone is more sufficient over $Ni(OH)_2$-SDS, which facilitates reduction of NiOOH to $Ni(OH)_2$

Supplementary Note 6

As displayed in Supplementary Fig. 21, the cyclohexanone in $Ni(OH)_2$-SDS is adsorbed by the dodecyl chain of SDS due to the hydrophobic interaction. As mentioned in the main text, the adsorption energy between cyclohexanone and $Ni(OH)_2$-SDS was calculated to be –0.46 eV. Referred to previous literature[7], the strength of a typical covalent bond is in the range of 1–10 eV, which is significantly larger than the adsorption energy between cyclohexanone and $Ni(OH)_2$-SDS, indicating that the strength for the adsorption between cyclohexanone and $Ni(OH)_2$-SDS is not as strong as a covalent bond.

Hence, we deduce that there is a weak intermolecular interaction between cyclohexanone and $Ni(OH)_2$-SDS. In addition, the adsorption energy between cyclohexanone and water (the solvent) was calculated to be –0.17 eV, originated from the hydrogen bond between the O atom in cyclohexanone and H atom in water. As a result, The differential adsorption energy of cyclohexanone in $Ni(OH)_2$–SDS and in solvent was calculated to be –0.29 eV, suggesting that cyclohexanone is possible to be released from $Ni(OH)_2$-SDS at room temperature, especially with external disturbance under the reaction conditions, such as the strong stirring and the large concentration gradient between the interlayer and the edge of the nanosheets. The concentration of cyclohexanone at the edge sites would be decreased to nearly zero in the reaction according to the normal pulse voltammograms results (Fig. 4).

Supplementary Note 7

To demonstrate that the adsorbed cyclohexanone can be continuously released

during the electrocatalytic process, we conducted the following experiments. As shown in Supplementary Fig. 22, the $Ni(OH)_2$-SDS electrode was immersed in a 0.5 M KOH solution with 0.4 M cyclohexanone, holding for 30 min to ensure cyclohexanone adsorption. Then the electrode was extracted, rinsed by DI water to remove cyclohexanone on the outer surface. We prepared two copies of the electrode for the following experiments. For Experiment I, the electrode was immersed in 0.5 M KOH (5 mL) under magnetic stirring for 15 min. Then the electrolyte was analyzed by ^{1}H NMR. For Experiment II, the electrode was immersed in an electrochemical cell containing 0.5 M KOH (5 mL), and electrooxidation reaction was performed at 1.5 V vs RHE for 15 min. Then the electrolyte was analyzed by HPLC.

By employing these two experiments, if cyclohexanone can be released from $Ni(OH)_2$ interlayer during the electrocatalytic process, cyclohexanone (Experiment I) and adipic acid (Experiment II) would be observed. To attain sufficient concentrations for analysis, both Experiments I and II were repeated 5 times before the final electrolytes were subjected to analysis. As a comparison, pure $Ni(OH)_2$ electrode was also treated following the similar procedures.

The results show that, for $Ni(OH)_2$-SDS, cyclohexanone was observed in Experiment I (determined by ^{1}H NMR), and adipic acid was observed in Experiment II (determined by HPLC), suggesting that the adsorbed cyclohexanone in $Ni(OH)_2$-SDS can be released during the electrocatalytic process. In contrast, the concentration of cyclohexanone and adipic acid were significantly lower by using pure $Ni(OH)_2$. We ascribe the observation of the signals over pure $Ni(OH)_2$ to the remaining of cyclohexanone on electrode surface.

Taken together, these results suggest that $Ni(OH)_2$-SDS can promote the adsorption of cyclohexanone, and the adsorbed cyclohexanone in $Ni(OH)_2$-SDS interlayer can be released during the electrocatalytic process.

Supplementary Note 8

The OH^- is involved in the process of cyclohexanone oxidation, which is proposed

to be activated on the NiOOH active sites at edge of the nanosheet to deliver surface-adsorbed oxygen species such as *OH under applied potential. The *OH shows electrophilic property which can react with nucleophiles derived by cyclohexanol/cyclohexanone. Since the active sites over $Ni(OH)_2$-SDS and $Ni(OH)_2$ for electrooxidation of cyclohexanone are mainly located at the edge of the nanosheet, we postulate that the intercalated SDS in the interlayer of $Ni(OH)_2$ may not seriously affect the diffusion process of OH^- from the bulk electrolyte to the edge of the nanosheet.

Supplementary Note 9

We ascribe the decrease of adipic acid formation rate to the reduced concentration of cyclohexanone in the reaction system. During the reaction, the electrolyte (0.5 M KOH with 50 mM cyclohexanone) in the glass bottle was continuously fed into the electrolyzer. Since the single-pass conversion of cyclohexanone was relatively low, the electrolyte was transported into the reactor multiple time by circulation. With the prolongation of the reaction time, the concentration of cyclohexanone in the reaction system was decreased, thus the formation rate of adipic acid was decreased accordingly.

The FE of $Ni(OH)_2$-SDS at each time point and the corresponding potential-time curve during the 24-hours flow cell test at 0.8 A have been provided in the Supplementary Fig. 36. The FE and formation rate of adipic acid were decreased gradually with the prolongation of the reaction time, whilst the reaction potential was increased, which are due to the decrease of cyclohexanone concentration in the electrolyte, thus mass transfer of cyclohexanone would be suppressed and OER would be more competitive.

Supplementary Note 10

We propose an electrocatalysis-coupling-electrodialysis (EC-ED) system might be helpful to separate adipic acid and simultaneously reuse the alkali, thus reducing the separation cost to a great extent. As illustrated in Supplementary Fig. 37 shown below,

the proposed ED device is composed of two pairs of acid and alkali chambers, separated by three bipolar membranes (BP) and two cation exchanges membranes (CEM). The electrooxidation reaction was conducted in a 0.5 M KOH in the EC device with the formation of AA^{2-}. The reaction effluent is then transported to the ED device. The CEM is used to separate K^+ ion in the electrolyte by electric field. At the same time, the bipolar membrane can split H_2O to OH^- and H^+ under electric field. As a result, H^+ would combine with AA^{2-} in the anode chamber to afford AA, which can be further concentrated and separated by traditional concentration methods (such as evaporation, anion exchange resins.[8] Meanwhile, OH^- would combine with K^+ in the cathode chamber to deliver KOH, which can pass through the electrocatalytic reactor multiple times for circulation. Based on this concept, we are setting up the EC-ED system for adipic acid separation in our lab

Supplementary Note 11

Model construction. The model of bulk $Ni(OH)_2$-SDS was constructed according to the X-ray diffraction pattern (Fig. 2b). The space group of $Ni(OH)_2$–SDS is R–3m:H, with the lattice parameters of $a = b = 2.82$ Å, $c = 29.5$ Å, $\alpha = \beta = 90°$, $\gamma = 120°$. The supercell of $Ni(OH)_2$-SDS used in the spin-polarized density functional theory calculations is 4 × 4 × 1 in the *a*, *b*, *c* directions. Since the polymorph of $Ni(OH)_2$-SDS is the alpha type, one sixteenth of H atoms in the hydroxyl of $Ni(OH)_2$ are removed so that the $Ni(OH)_2$ matrix is cationic. Two dodecylsulfate anions were put into the interlayer space of $Ni(OH)_2$ to make the model neutral according to the molar ratio of Ni : dodecylsulfate in experiment. Thus the chemical formula of $Ni(OH)_2$-SDS is $Ni_{16}(OH)_{30}(C_{12}H_{25}SO_4)_2$.

The model of solvent was constructed by putting 125 H_2O molecules in a solvent box with the lattice parameters of $a = b = c = 15.75$ Å, $\alpha = \beta = \gamma = 90°$. The density of this solvent box is about 1 g·cm^{-3}. The chemical formula of the solvent box is $H_{250}O_{125}$.

Computational methods. The spin-polarized density functional theory calculations

in this work was performed with the Cambridge Sequential Total Energy Package.[9] The spin-polarized density functional theory calculations were performed with the plane wave implementation at the level of generalized gradient approximation Perdew–Burke–Ernzerhof.[10] The ionic core of Ni was described by the ultrasoft pseudopotentials to reduce the number of plane waves needed to expand the Kohn–Sham equations.[11] The cutoff energy was set as 400 eV and the k-point meshes were set as 3 × 3 × 1. The adsorption energy E_B between cyclohexanone and $Ni(OH)_2$-SDS was calculated with equation (1):

$$E_{\mathrm{B}} = E_{\mathrm{cyclohexanone\,\&\,Ni(OH)_2-SDS}} - E_{\mathrm{cyclohexanone}} - E_{\mathrm{Ni(OH)_2-SDS}} \quad (1)$$

where $E_{\mathrm{cyclohexanone\,\&\,Ni(OH)2-SDS}}$, $E_{\mathrm{cyclohexanone}}$, and $E_{\mathrm{Ni(OH)2-SDS}}$ represent the energies of $Ni(OH)_2$–SDS adsorbed with cyclohexanone, cyclohexanone, and $Ni(OH)_2$–SDS, respectively. The adsorption energy between cyclohexanone and solvent was calculated in the same way.

Supplementary References

1. Karimi Estahbanati, M.R. *et al.* Selective photocatalytic oxidation of cyclohexanol to cyclohexanone: A spectroscopic and kinetic study. *Chem. Eng. J.* **382**, 122732 (2020).
2. Lyalin, B. V. & Petrosyan, V. A. Electrosynthesis of adipic acid by undivided cell electrolysis. *Russ. Chem. Bull.* **53**, 688-692 (2004).
3. Nong, H.N. *et al.* Key role of chemistry versus bias in electrocatalytic oxygen evolution. *Nature* **587**, 408-413 (2020).
4. Lyalin, B. V. & Petrosyan, V. A. Electrosynthesis of glutaric acid and regularities of electrocatalytic oxidation of cycloalkanones at a NiOOH anode in aqueous NaOH. *Russ. Chem. Bull.* **58**, 2426-2431 (2009).
5. Zhou, H. *et al.* Selectively Upgrading Lignin Derivatives to Carboxylates through Electrochemical Oxidative C(OH)-C Bond Cleavage by a Mn-Doped Cobalt Oxyhydroxide Catalyst. *Angew. Chem. Int. Ed.* **60**, 8976-8982 (2021).
6. Deng, X. *et al.* Understanding the Roles of Electrogenerated Co(3+) and Co(4+) in Selectivity-Tuned 5-Hydroxymethylfurfural Oxidation. *Angew. Chem. Int. Ed.* **60**, 20535-20542 (2021).
7. Illas, F. Fundamental Concepts in Heterogeneous Catalysis. By Jens K. Nørskov, Felix Studt, Frank Abild-Pedersen and Thomas Bligaard. *Angew. Chem. Int. Ed.* **54**, 10404-10405 (2015).
8. Zeidan, H. & Marti, M.E. Separation of Formic Acid from Aqueous Solutions onto Anion Exchange Resins: Equilibrium, Kinetic, and Thermodynamic Data. *J. Chem. Eng. Data* **64**, 2718-2727 (2019).
9. Clark, S. J. *et al*. First principles methods using CASTEP. *Z. Kristallogr.* **220**, 567–570 (2005).
10. Perdew, J. P., Burke, K. & Ernzerhof, M. Generalized gradient approximation made simple.

Phys. Rev. Lett. **77**, 3865–3868 (1996).
11. Vanderbilt, D. Soft self-consistent pseudopotentials in a generalized eigenvalue formalism. *Phys. Rev. B Condens. Matter.* **41**, 7892-7895 (1990).
