# Supplementary material for: Electrocatalytic synthesis of adipic acid coupled with H2 production enhanced by a ligand modification strategy
Source: Nat Commun. 2022 Aug 25;13:5009. doi: 10.1038/s41467-022-32769-0 (PMC9411531; doi:10.1038/s41467-022-32769-0)
Supplement: Supplementary file 2 — Description of Additional Supplementary Files [file 41467_2022_32769_MOESM2_ESM.pdf]

Description of Additional Supplementary Files

File Name: Supplementary Movie 1
Description: The total time sequence of cyclohexanone diffusion in $Ni(OH)_2$.

File Name: Supplementary Movie 2
Description: The total time sequence of cyclohexanone diffusion in $Ni(OH)_2$-SDS.
